# Making Large-Scale Networks from fMRI Data

**DOI:** 10.1371/journal.pone.0129074

**Published:** 2015-09-01

**Authors:** Verena D. Schmittmann, Sara Jahfari, Denny Borsboom, Alexander O. Savi, Lourens J. Waldorp

**Affiliations:** 1 Department of Methodology and Statistics/Social and Behavioral Sciences, Tilburg University, Tilburg, the Netherlands; 2 Department of Cognitive Psychology, Vrije Universiteit, Amsterdam, the Netherlands; 3 Psychological Methods/Social and Behavioral Sciences, University of Amsterdam, Amsterdam, the Netherlands; Hospital for Sick Children, CANADA

## Abstract

Pairwise correlations are currently a popular way to estimate a large-scale network (> 1000 nodes) from functional magnetic resonance imaging data. However, this approach generally results in a poor representation of the true underlying network. The reason is that pairwise correlations cannot distinguish between direct and indirect connectivity. As a result, pairwise correlation networks can lead to fallacious conclusions; for example, one may conclude that a network is a small-world when it is not. In a simulation study and an application to resting-state fMRI data, we compare the performance of pairwise correlations in large-scale networks (2000 nodes) against three other methods that are designed to filter out indirect connections. Recovery methods are evaluated in four simulated network topologies (small world or not, scale-free or not) in scenarios where the number of observations is very small compared to the number of nodes. Simulations clearly show that pairwise correlation networks are fragmented into separate unconnected components with excessive connectedness within components. This often leads to erroneous estimates of network metrics, like small-world structures or low betweenness centrality, and produces too many low-degree nodes. We conclude that using partial correlations, informed by a sparseness penalty, results in more accurate networks and corresponding metrics than pairwise correlation networks. However, even with these methods, the presence of hubs in the generating network can be problematic if the number of observations is too small. Additionally, we show for resting-state fMRI that partial correlations are more robust than correlations to different parcellation sets and to different lengths of time-series.

## Introduction

In recent years, the use of network science for investigating connectivity in the brain from functional magnetic resonance imaging (fMRI) has brought about some amazing results [1–3]. For instance, the functional brain network appears to have a scale-free connectivity structure [4], which implies the existence of a small number of hubs (i.e., nodes with disproportionally numerous connections); intelligence seems to correlate negatively with average pathlength (i.e., average number of steps of shortest paths between each node pair) in the functional brain network [5]; and children and young-adults have similar small-world brains [6]. Small-world networks exhibit high local clustering (i.e., interconnectedness in neighborhoods of nodes) and low average pathlengths compared to equidimensional random networks [7].

**Fig 1:**
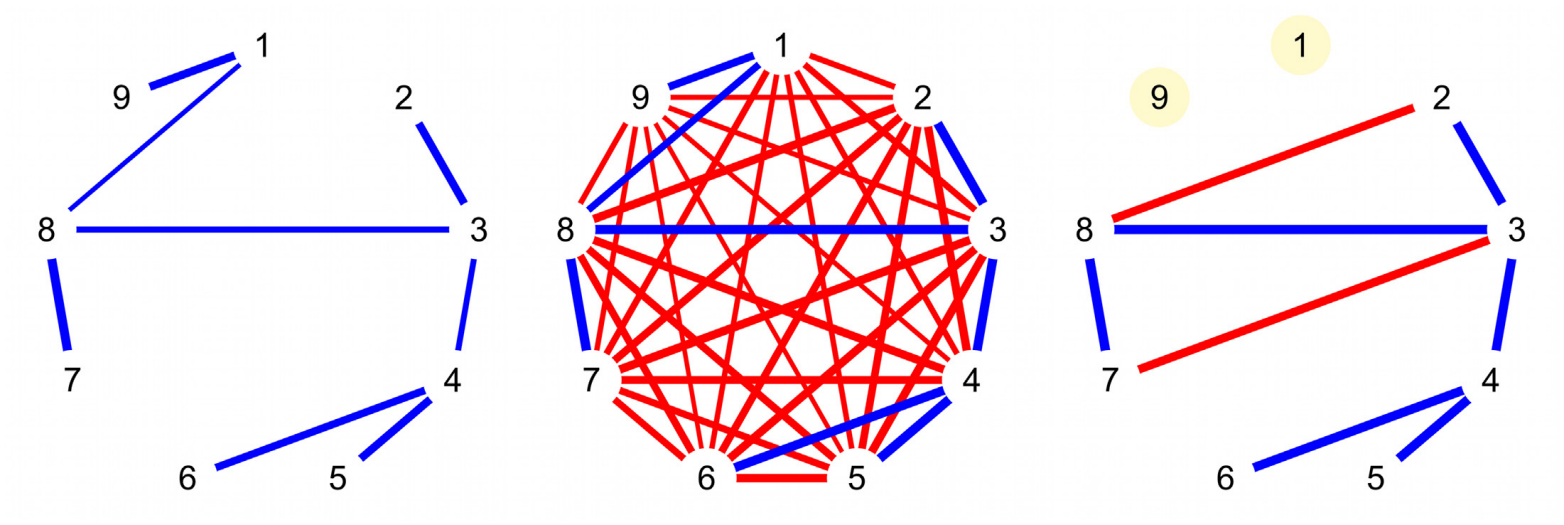
Illustration of pairwise vs partial correlation networks. Thicker edges represent stronger absolute correlations. Left: true network of partial correlations (blue), with 8 connections, no triangles. Middle: associated pairwise correlation network, with erroneous direct connections (red) that form 84 triangles. Right: pruned network of 8 strongest pairwise correlations, with two isolated nodes (yellow) and two erroneous connections (red) that form 2 triangles (2-3-8 and 3-7-8). Comparing the true partial correlation network on the left with the pruned pairwise correlation network on the right, which consists of the same number of edges as the underlying network, three differences stand out. Firstly, indirect connections may appear as direct connections (i.e., nodes 2–8 and nodes 3–7). This results in an excessive number of triangles, affecting network measures such as small-worldness. Secondly, while the true network is connected (i.e., there exists a path between each pair of nodes), pruned pairwise correlation networks tend to consist of isolated (groups of) nodes (i.e., nodes 1 and 9). Thirdly, the number of connections of a node may differ from the true number of connections (e.g., node 3 has four instead of three edges). In larger networks, hub nodes may emerge erroneously.

Functional brain networks are frequently inferred from pairwise correlations, assuming they identify true functional connectivity if they pass some threshold [2–4, 8, 9]. A pairwise correlation that exceeds this threshold may arise from a direct connection; however, it may also be spurious. As illustrated in Fig 1, correlations may result from indirect connections. This may lead to an excess of triangles (completely connected triples of nodes) in the network (e.g., [10, 11]). This observation has important ramifications for the validity of network analyses in fMRI data, because triangles of connected nodes feature in network metrics, such as small-worldness. If using pairwise correlations leads to spurious relationships, these may negatively affect subsequent network analyses and substantive conclusions (e.g., erroneously concluding that the network has a small-world topology, or that its connectivity structure is scale-free when it is not).

**Fig 2:**
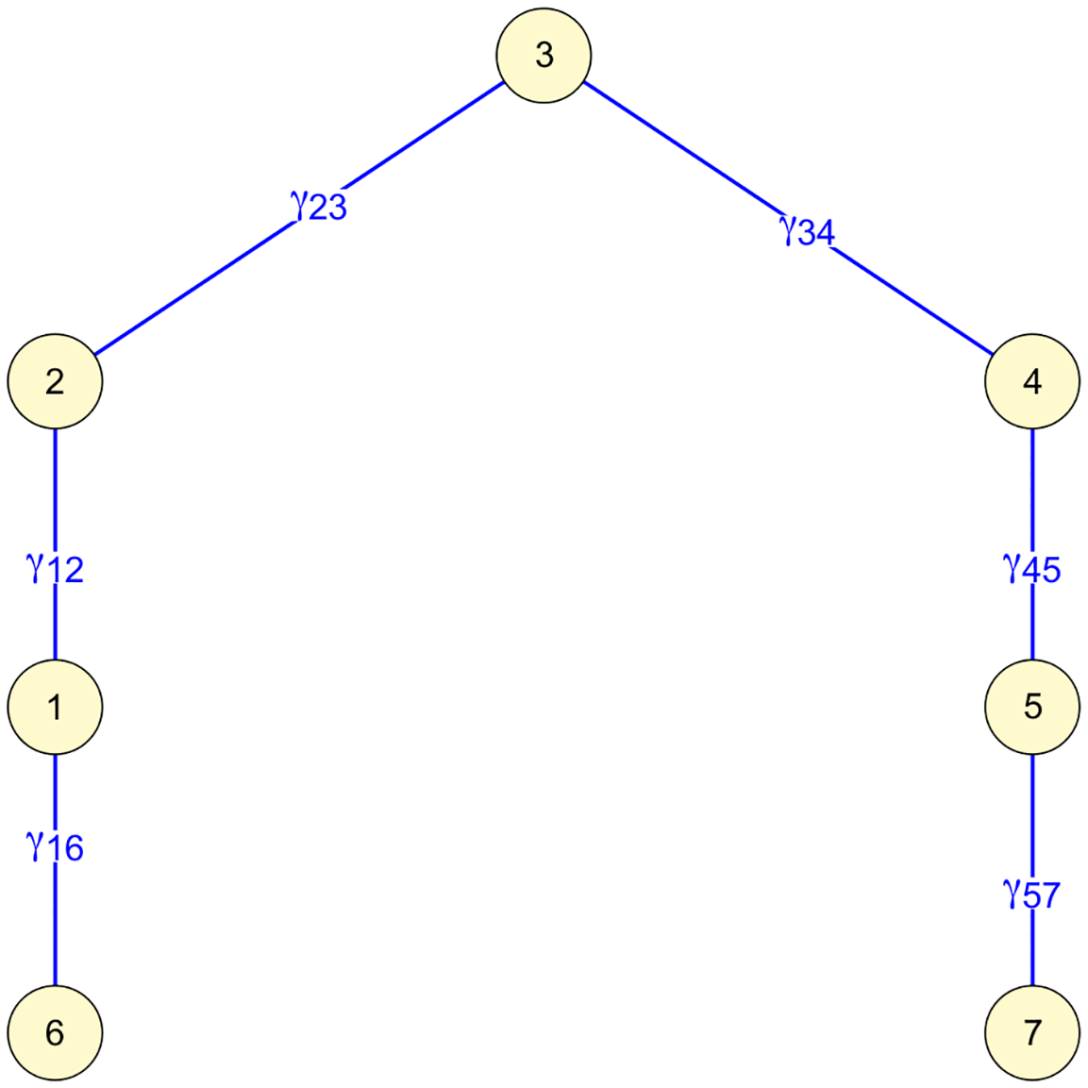
Exemplary network with path from node 1 to 5, showing partial covariances *γ*_*ij*_.

The correlation (or the unscaled version, the covariance) can be considered as a function of the partial correlations (partial covariances). Consider the network in Fig 2 and suppose that this is the true underlying network. Here is a path from 1 to 5 as 1 − 2 − 3 − 4 − 5. For Gaussian variables the covariance is a function of the product of partial covariances *γ*_12_ *γ*_23_ *γ*_34_ *γ*_45_ [12, 13]. Because of this the correlation between nodes 1 and 5 is nonzero. It also follows that partialling out (i.e., conditioning on) any or all of the nodes in the path is sufficient to obtain the correct interpretation that there is no direct connection between nodes 1 and 5. In general, there is no knowledge of which paths there are, and so it seems best to condition on all other nodes.

For networks with small (up to 50) numbers of regions, several inference methods have been proposed and compared in small-world-type networks, suggesting superior performance of methods that involve the estimation of partial correlations [14]. Pairwise correlation performed a little less well in typical scenario’s, which was attributed to the ability of partial correlation methods to distinguish direct connections [14]. In all scenario’s that were investigated in [14], the number of observations *n* (at least 50 observations) was equal to or larger than the number of regions *p* (at most 50 regions). Also for the case in which the number of observations *n* is larger than the number of regions *p* (i.e., *p* < *n*), novel modeling and inference methods to obtain a network connectivity structure have been proposed in recent studies [15–20]. This case thus receives considerable attention in the literature. In contrast, the question of how the methods fare in the case where the number of regions is large (thousands of regions), yet the number of observations is smaller than the number of regions (i.e., *n* < *p*) has not been systematically addressed so far in the context of brain networks. Nevertheless, pairwise correlation is commonly being used to infer large-scale fMRI networks from small sample sizes [2–4, 8, 9]. In this paper we address the need of a systematic comparison of the performance of methods to determine a large-scale functional brain network. We consider partial correlations as an alternative to pairwise correlation [21]. Computing partial correlations directly requires more observations than number of regions, which is not feasible for large-scale networks. Therefore, we consider three different estimators for partial correlations, the graphical lasso [22], ridge regression [23], and the shrinkage estimator [24, 25]. Additional methods that were considered by [14] and developed for the *p* < *n* case, like causal inference methods, are not included here, because they are not suitable if the number of nodes exceeds the number of observations.

To investigate the accuracy of pairwise and partial correlation estimators on large-scale networks we created four different network topologies: a random network [26], a small-world network [7], a network with hubs [27], and a small-world network with hubs [28]. We hypothesize that using pairwise correlations results in a poor representation of the true network, i.e., metrics, like small-worldness, betweenness centrality, and other metrics will be inaccurate. Furthermore, we hypothesize that partial correlations will provide a reasonable representation of the true large-scale network, and consequently many network metrics will be accurate. Additionally, we compare networks based on pairwise and partial correlations from fMRI resting-state data of different sample sizes and spatial resolutions.

## Materials and Methods

In this paper, we analyzed simulated data and fMRI resting-state data (deposited at Data Archiving and Networked Services—DANS, http://persistent-identifier.nl/?identifier=urn:nbn:nl:ui:13-okb6-1d). We generated and analyzed all networks using R [29]. As explained in the following sections, we used partial and pairwise correlations in order to generate the data, and again in the subsequent inference of the network topologies. This might evoke the impression that we adapted the data generation process to one of the inference methods. However, the opposite is true. We generated the data based on network theory. In particular, the connections in a network can be described as a set of conditional independence relations. For Gaussian data, these independence relations are represented in the partial correlation matrix of a network, while the observed correlations between activity of pairs of nodes are captured in the correlation matrix of a network [13]. Our choice of the inference methods includes the commonly used method of pairwise correlations, and three other partial correlation methods, which are more suitable based on network theory.

### Inference of Networks

To infer a network structure, that is, to determine the connections in the network, we require an estimate of the values of the edges. Such an estimate can be obtained by computing pairwise correlations or partial correlations. Pairwise correlations can always be computed for Gaussian data. This is, however, not true for the partial correlations.

If the number of observations is larger than the number of regions (nodes) in the required network (i.e., *p* < *n*), then the sample covariance matrix can be used to compute the partial correlations [13]. Let *Y*_*i*_ denote the *p*-variate vector for all regions of volume (time point) *i* = 1,2,…,*n*, and let $\bar{Y}$ denote the average over the time points. Then the sample covariance matrix *S*, from which the correlations and partial correlations are computed, equals [13]

$$S=\frac{1}{\left(n-1\right)}\sum_{i=1}^{n}\left(Y_{i}-\bar{Y}\right){\left(Y_{i}-\bar{Y}\right)}^{\prime }$$ (1)

The partial variances, covariances, and correlations can be obtained from the concentration matrix Γ, which is the inverse of *S*. The partial correlations are computed by multiplying the off-diagonal elements of Γ with −1 and dividing by the square root of the respective diagonal elements of Γ, that is, the partial correlation between nodes *i* and *j* equals

$$\frac{-\gamma_{ij}}{\sqrt{\gamma_{ii}\gamma_{jj}}}.$$ (2)

The step of inverting matrix *S* requires that the matrix *S* be positive definite, that is, that the rank of the space implied by *S* is the same as its dimension *p*, which holds if *n* > *p* [13]. If, however, the number of time points *n* is smaller than the number of regions *p*, *n* < *p*, then we cannot use *S* directly and we need to add information about the structure of Σ, the true covariance matrix representing the network. The methods to compute partial correlations when *p* < *n* commonly impose information about the sparsity (low number of edges) in the network. We selected the following three different methods to do so.

#### Partial Correlation by Shrinkage Estimation

The shrinkage estimator ${\hat{\Sigma }}_{S}$ is obtained by a linear combination of the maximum likelihood (ML) estimate *S* of the covariance matrix and a specified target matrix *T*, as follows

$${\hat{\Sigma }}_{S}=\left(1-\lambda_{s}\right)S+\lambda_{s}T$$ (3)

*T* here is a matrix with the variances in *S* on the diagonal and 0 on the off-diagonal. The parameter 0 ≤ *λ*_*s*_ ≤ 1 is estimated from the data. See Schäfer and Strimmer [24] for more details, also for the function *pcor.shrink* in R to compute the shrinkage estimate.

#### Partial Correlations by Moore-Penrose Inverse (Ridge Regression)

A Moore-Penrose inverse of a covariance matrix *S* is defined by [30]

$$S^{+}=\lim_{\lambda \to 0}{\left(S^{\prime }S+\lambda_{r}I\right)}^{-1}S^{\prime }$$ (4)

where *I* is the identity matrix, and *λ*_*r*_ ≥ 0 is the regularization parameter. We used the function *ginv* in R to calculate the Moore-Penrose inverse. The equivalent ridge regression version which also includes adjusted degrees of freedom can be found in Hoerl and Kennard [23].

#### Partial Correlations by Graphical Lasso Inverse

The graphical lasso estimate of the inverse covariance matrix Σ^−1^ is defined as the maximum of the penalized log-likelihood function

$$\log|\Sigma^{-1}|-\text{tr}\left(S\Sigma^{-1}\right)-\lambda_{l}\left|\right|\Sigma^{-1}{\left|\right|}_{1}$$ (5)

where *S* is the sample covariance matrix, ∣*A*∣ is the determinant of matrix *A*, tr denotes the trace of a matrix, and ∣∣*A*∣∣_1_ = ∑_*ij*_∣*a*_*ij*_∣ is the sum of the absolute values of the matrix *A* [22]. Maximization is performed among symmetric, positive definite matrices. We used the R-package *glasso* [31] to estimate the partial correlations. For each data-set, the parameter *λ*_*l*_ ≥ 0 was determined separately in such a way that the method resulted in networks with a predefined set of proportion of edges, as described in the next section.

### Selection of Connections

The four methods above result in full networks, in which each possible connection has a certain estimated weight (strength). From these full networks, we selected the connections with the largest absolute weights, and other connections were removed (i.e., their weight was set to 0). From each of the full networks, we arrived at three pruned networks, differing in the number of selected connections: (a) a network with the same proportion of edges as the generating network (e.g., if the generating network consisted of 10000 edges, we selected the 10000 connections with the strongest absolute estimated weights), (b) a network with 20% too few connections (e.g., if the generating network consisted of 10000 edges, we selected the 8000 connections with the strongest absolute estimated weights), and (c) a network with 20% too many connections (e.g., if the generating network consisted of 10000 edges, we selected the 12000 connections with the strongest absolute estimated weights). This procedure ensures that comparing connectivity for each of the four methods is based only on differences in the estimators and is not confounded by selection procedures.

### Network Characteristics

R and the contributed packages *igraph* [32] and *qgraph* [33] were used to calculate the following network characteristics of interest and to graphically display networks. Average path length, that is, the average number of steps of the shortest paths between each node pair, was calculated with function *average.path.length* in *igraph*. Average degree is simply the average number of connections of a node in the network.

The global clustering coefficient [34] we employed, considered the degree, to which the nodes’ neighbors (i.e., the nodes to which a node is directly connected) are also interconnected. It reflects the proportion of triangles in the network, ranging from 0 (i.e., if the network does not contain triangles) to 1 (i.e., if each two neighbors of all nodes are directly connected as well). The clustering coefficient was calculated with function *transitivity(, type = “global”)* in *igraph*. Local transitivity, reflecting the proportion of triangles around individual nodes, was determined for ROIs in the resting-state fMRI data using function *transitivity(, type = “local”)* in *igraph*.

The small-worldness index, as proposed by Humphries and Gurney [35], is based on a trade-off of high clustering and short average path lengths, each in relation to a random network of the same size. It is calculated as the ratio of the clustering coefficient of the network divided by the expected clustering coefficient of a random network, and the average path length of the network divided by the expected average path length of a random network. By definition, random networks have an index close to 1, and the higher the index, the more pronounced the small-worldness structure of the network.

The networks from which we generated the data all consisted of a single component, that is, every node is either directly or indirectly connected to any other node in the network. This is not necessarily the case in the estimated networks, where different sets of nodes may turn out to be unconnected to another. The number and size of the components (i.e., connected sets of nodes) were determined using function *clusters* in *igraph*.

Finally, average betweenness centrality was calculated as the average of the number of shortest paths on which a node lies, which was obtained using function *betweenness* in *igraph*.

### Data Simulation

**Fig 3:**
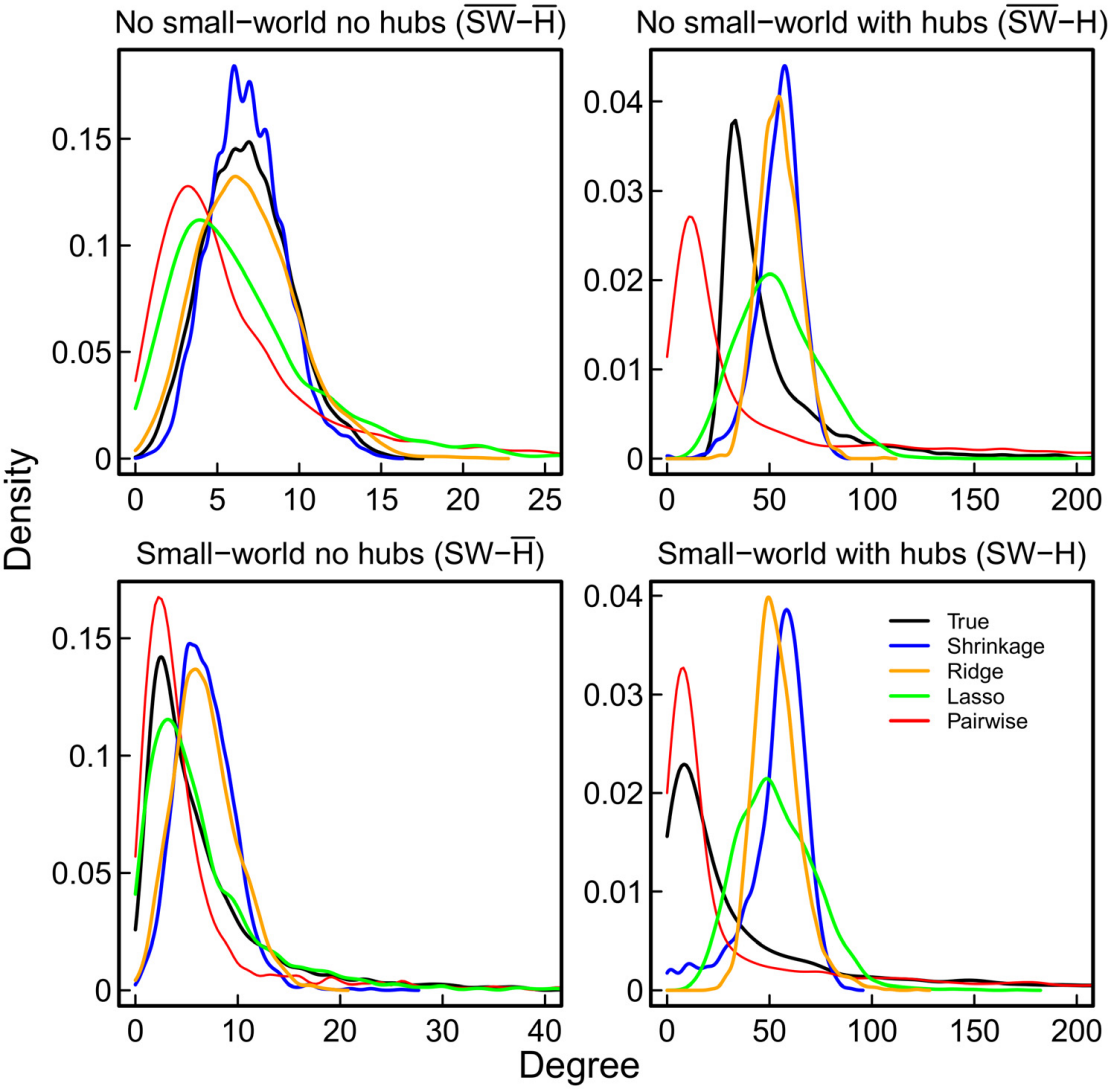
Recovery of degree distributions based on 500 observations. Densities of the true (black) and recovered node degrees of shrinkage (blue), ridge (orange), and lasso (green) estimated partial correlations, and of pairwise correlations (red). NB: x-axis cut off.

In order to compare the inference methods in different relevant scenario’s, we generated four network topologies of 2000 nodes each that differed in the degree distribution and small-worldness [34]. Black lines in Fig 3 show the degree distributions of these network topologies. These four different network topologies featured a small-world structure (SW) or not ($\bar{\text{SW}}$, random network), and contained hubs (H) or not ($\bar{\text{H}}$). In order to match empirically found brain network densities (i.e., proportion of edges), these networks were designed to be sparse (around 3% of possible edges; as found by [36]) or very sparse (around 0.3% of possible edges; similar to [37]). Nevertheless, due to the huge number of possible edges in a network with *p* = 2000 nodes (*p* × (*p* − 1)/2 = 1999000), this corresponded to approximately 54000 and 6800 edges for the sparse and very sparse networks, respectively.

As explained in detail below, an autoregressive time-series of length 500, 1000, 3000, and 10000 was produced for each node in each network. In covariance estimation, a ratio of observations *n* to the number of variables *p* of about 15 is typically desirable, but here we have a much smaller ratio, indicating the *n* ≪ *p* scenario. With *p* of 2000 nodes, and *n* of 500, 1000, 3000, or 10000 observations, the *n*/*p* ratio would range between.25 to 5. However, due to the autocorrelation of the time-series, these observations were not independent of each other. This implies that the effective number of observations was even smaller. Correcting for the autocorrelation in the time-series *ρ*, we arrive at the effective numbers of observations $n^{\prime }=n\times \frac{1-\rho }{1+\rho }$ of 166.7, 333.3, 1000, and 3333.3 [38]. The effective *n*′/*p* ratio is thus lower, ranging from 0.083 to 1.667.

Data simulation consisted of three steps: First, we generated network topologies that differed according to degree distribution and small-worldness. Secondly, we sampled weighted networks for each of these network topologies. Thirdly, we sampled time-series data for each of the weighted networks. In the next subsections, these steps are described in detail.

Step 1: Generation of Network Topologies

Two small-world networks were built using an algorithm from social networks [28], which, in each iteration adds certain connections, and with probability *p*_*d*_ removes certain connections, and which, depending on the value of *p*_*d*_, will lead to small-world networks with or without hubs. The exact algorithm is described in detail by Davidsen et al. [28]. We employed the algorithm with 2000 nodes, using 1250000 iterations to build each network. To obtain a small-world network with hubs SW-H and one without hubs $\text{SW}-\bar{\text{H}}$, parameter *p*_*d*_ of the algorithm was set to.008 and.1, respectively. These parameter values were chosen, because they produced networks with the desired properties. The next network, containing hubs without small-world structure $\bar{\text{SW}}-\text{H}$, was generated using a linear preferential attachment algorithm discussed by [27], as implemented in the function *barabasi.game* in Rpackage *igraph* [32]. As this algorithm could result in networks with more than one edge between two nodes, and with an edge from a node to itself, such improper connections were then removed with the *simplify* function in *igraph* to arrive at a viable network. The number of nodes was set to 2000, and the number of edges to add in each time step, *m*, was set to 29. This value of *m* was chosen, because it resulted in a network comparable to SW-H with respect to density. A random network without small-world structure and without hubs $\bar{\text{SW}}-\bar{\text{H}}$ was generated with 2000 nodes and density .003 by random sampling of edges, in which each possible edge had the same probability of .003 of being present. For post hoc comparison, a complementary random network with density.03 and 2000 nodes was generated analogously $\bar{\text{SW}}-\bar{\text{H}}-\text{c}$. To ensure connectedness of all networks, a few isolated nodes were removed. To arrive at representative network topologies, we generated 100 networks for each network type, and selected the network that had the smallest or next-to-smallest normalized Euclidian distance from the respective group mean of transitivity, average path length, average degree, variance of degrees, average betweenness centrality, and small-worldness. The resulting network sizes and other network characteristics of interest are shown in Table 1. Each generated network topology was represented as an adjacency matrix, in which the presence of a connection between a row-node and a column-node is indicated by entry 1, and the absence of this connection is indicated by entry 0. From these adjacency matrices, we generated weighted networks as follows.

**Table 1:** Characteristics of simulated networks.

	$\bar{\text{SW}}-\bar{\text{H}}$	$\text{SW}-\bar{\text{H}}$	SW-H	$\bar{\text{SW}}-\text{H}$	$\bar{\text{SW}}-\bar{\text{H}}-\text{c}$
Number of nodes *p*	1998	1982	2000	2000	2000
Number of edges	6843	6744	53748	54720	53581
Prop. of edges	0.003	0.003	0.03	0.03	0.03
Avg. path length	4.17	4.16	2.48	2.11	2.21
Clustering coefficient	0.00	0.16	0.29	0.07	0.03
Small-worldness	1.02	45.86	10.02	2.79	1.03
Avg. degree	6.85	6.80	53.75	54.72	53.58
Min. degree	1	1	1	26	31
Max. degree	16	72	573	886	78
Avg. betweenness	3161	3135	1475	1111	1204
Avg. strength	2.06	1.60	2.59	2.58	4.28

Step 2: Generation of Weighted Networks

The weighted networks we use can be represented as a partial correlation matrix, where each zero represents conditional independence [13]. We constructed a partial correlation matrix *R* by drawing values from the uniform distribution *U*([−1,−.01]∪[.01,1]), one for each edge, to arrive at the (possibly singular) partial correlation matrix *R*_*s*_, which has ones on the diagonal, and sampled values on those off-diagonal positions where the adjacency matrix equals 1. We then regularized *R*_*s*_ to have the matrix represent a distribution with dimension 2000 (i.e., the resulting matrix is positive definite), and reset those off-diagonal elements, where the respective adjacency matrix equals 0, to 0 to ensure that weights of absent edges are exactly zero. If this step is ignored, the resulting matrix *R* is not a proper representation of the true network. The resulting matrix is the partial correlation matrix *R*. The partial correlation matrix contains the weights of the connections on the off-diagonal. Table 1 shows the average strength (weighted degree) [39] of the nodes in the weighted networks. For all four partial correlation matrices we calculated a correlation matrix *C* by multiplying the off-diagonal elements of *R* with −1, and then calculating the pseudo-inverse using the function *pcor2cor* of the R-package *corpcor* [40]. We then multiplied the correlation matrix *C* by a uniform variance of 2, to arrive at a positive definite covariance matrix Σ for each of the four different networks.

Step 3: Generation of Time-Series Data

From the covariance matrices Σ, we generated time-series data with an AR(1) temporal structure, which is an appropriate lag for preprocessed fMRI data [41] [42, 43]. The time-series data of length 10000 were constructed by first sampling 10000 random values for each node from a standard normal distribution with mean zero and variance 1, collected in *Z* (*N* × 10000-dim. matrix). We then pre-multiplied *Z* with the transpose of the Cholesky decomposition of Σ, and post-multiplied the resulting matrix with the Cholesky decomposition of the Toeplitz matrix of an AR(1) process with autoregressive parameter *ρ* = .5. From each of the resulting full data matrices, we built 4 (nested) datasets: the first 500 timepoints, the first 1000 timepoints, the first 3000 timepoints and all 10000 timepoints.

### Magnetic Resonance Imaging Scanning Procedure

The fMRI resting-state data were acquired in a single scanning session on a 3T scanner (Philips). For the resting-state protocol participants were instructed to stay alert and focus on a white fixation cross; presented on a black-projection screen that was viewed via a mirror system attached to the magnetic resonance imaging (MRI) head coil. In total, 240 T2*-weighted echoplanar images (EPIs) (2202 mm FOV; 962 in plane resolution; 3.3 mm slice thickness; 0 mm slice spacing; TR 2000 ms; TE 28 ms; FA 90o, ascending orientation) were scanned. For registration purposes, a three-dimensional T1 scan was acquired before functional runs of an independent fMRI study (T1; TFE 218x226 mm FOV; 2562 in plane resolution; 182 slices, 1.2 mm slice thickness, TR 9.56 ms, TE 4.6 ms, FA 8, coronal orientation).

### Preprocessing of Resting-State fMRI Data

Preprocessing of the resting-state fMRI data was carried out using FEAT (FMRI Expert Analysis Tool) Version 5.98, part of FSL (FMRIB’s Software Library, www.fmrib.ox.ac.uk/fsl). The following pre-processing steps were applied; motion correction using MCFLIRT [44]; slice-timing correction using Fourier-space time-series phase-shifting; non-brain removal using BET [45]; grand-mean intensity normalization of the entire 4D dataset by a single multiplicative factor; highpass temporal filtering (Gaussian-weighted least-squares straight line fitting, with sigma = 50.0s).

### Parcellations of Resting-State fMRI Data

The parcellation procedure relied on a recently published structural segmentation procedure using the Desikan labeled mesh in freesurfer [46], [47]. More specifically, the Lausance 2008 parcellation within the Connectome viewer toolkit (http://www.cmtk.org) was used to create the 5 embedded hierarchical cortical parcellations within Freesurfer [36, 46, 48, 49]. This means that for each subject, the T1-weighted image is first segmented into 68 atlas based cortical parcels, using the freesurfer Desikan labled mesh from an average brain [47]. With the use of the Lausanne 2008 template (available in the connectome viewer toolkit), each parcel is then subdivided into smaller ROIs of approximately 1.5 cm^2^ to obtain the high resolution parcellations of 1000 ROIs. The 1000 cortical ROIs are then grouped into bigger ROIs to arrive at 5 separate parcellations with respectively 68, 114, 219, 448, and 1000 ROIs [46].

### Extraction of Time-Series of Resting-State fMRI Data

For each individual, all segmentations were transformed and registered onto the fMRI resting-state images. To obtain, the most refined transformation matrix, EPI images were first registered onto the individual T1 scan. The inverse of this matrix, per subject, was then used to register all T1 mapped segmentations into epi space. Consequently, the averaged times series across voxels was extracted per ROI, for each segmentation. Prior to the computations of networks, for each segmentation, the mean cerebral fluid and white matter signals were regressed from each time series. Note that, all time series were extracted from ROIs registered to individual EPI space.

### Participants

Data was collected from five healthy adults (mean age 24.8 years, range 21–32 years; 4 females). In accordance with the declaration of Helsinki, all participants provided written consent before the scanning session. The ethics committee of the Department of Developmental Psychology of the University of Amsterdam approved the experiment (approval number 2010-DP-1131) and all procedures complied with relevant laws and institutional guidelines. All participants were right handed and had normal or corrected-to-normal vision. A small part of the resting state fMRI data have been used for illustrative purposes in a different paper on model selection [50].

## Results

To give a complete picture of how the estimated networks differ by the four methods, we provide a combination of several network characteristics, and false and true positive rates (i.e., the probability of inferring an edge where there is none and the probability of recovering an existing edge, respectively). We first present the results of the following four networks: small-world structure with hubs (SW-H), small-world structure without hubs ($\text{SW}-\bar{\text{H}}$), hub network without small-world structure ($\bar{\text{SW}}-\text{H}$, and the sparser random network without small-world structure and without hubs ($\bar{\text{SW}}-\bar{\text{H}}$). The results of the complementary random network ($\bar{\text{SW}}-\bar{\text{H}}-\text{c}$) are presented in a post hoc comparison, as the results of the two random networks were comparable.

As mentioned above, we evaluate performance of the methods in the scenarios with the correct number of edges and nodes [51]. Also, we investigate performance when up to 20% below or above the true number of edges are selected. Fixing the number of connections to a certain number (fixed density) is directly related to choosing a certain cutoff threshold in estimated values or significance level [8]. This ensures that comparing connectivity for each of the four methods is based only on how a connection is made. That is, if a connection is judged to be present according to the pairwise correlation method, but absent according to the partial correlation method, this difference is exclusively due to the difference in estimators.

### Small-Worldness and Related Network Characteristics

**Fig 4:**
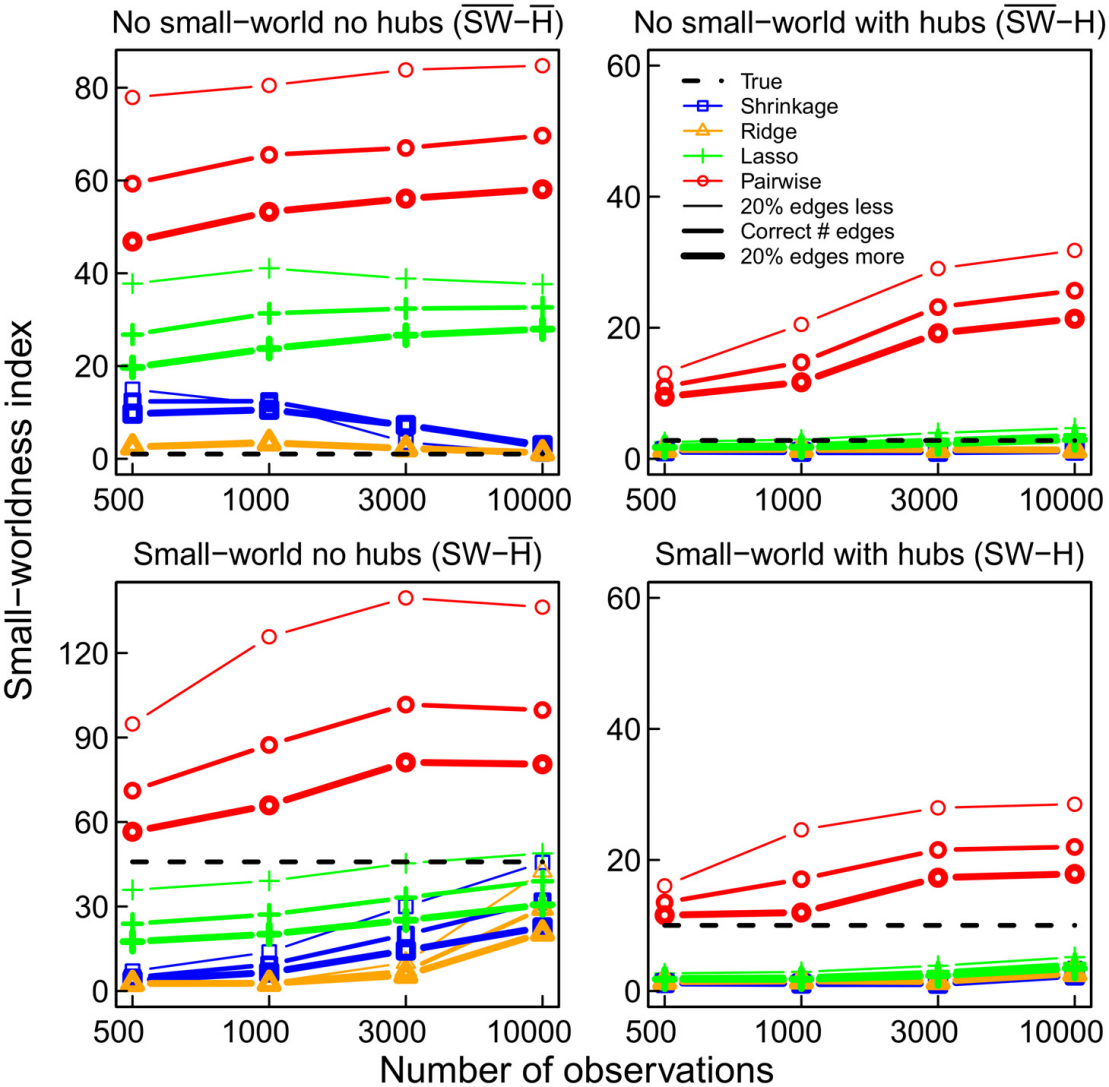
The small-worldness index for the four networks and the four estimation methods pairwise correlations (red), lasso (green), ridge (orange), and shrinkage (blue), compared to the true value −− (black). The thickness of the line represents the number of selected edges. Pairwise correlation networks always overestimate the small-worldness.

**Fig 5:**
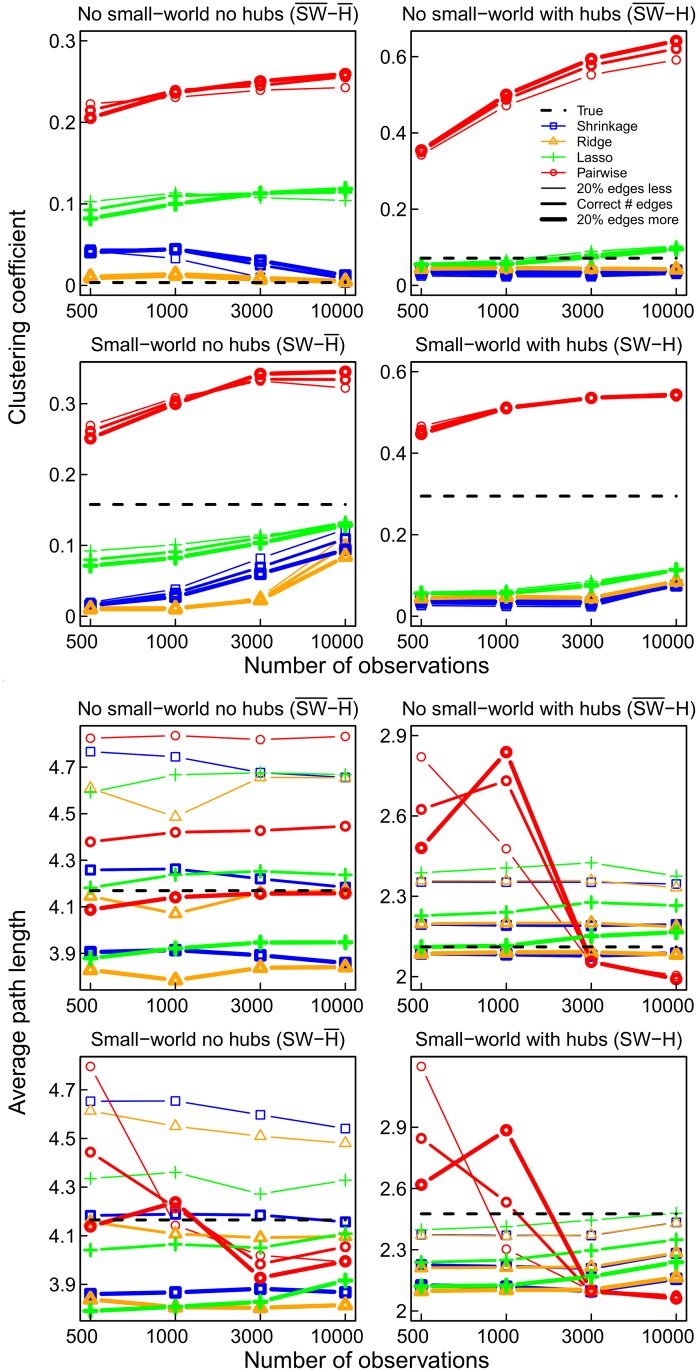
Clustering coefficient (upper) and average pathlength (lower) for the four networks and estimation methods.

As mentioned above, small-world networks are characterized by short average pathlengths and high clustering. This implies a high connectivity in each neighborhood of nodes [7, 35, 51]. Formally, the small-worldness index can be defined by the ratio of the clustering coefficient and the average pathlength relative to a random network of the same dimensions [35]. The small-worldness index of a network depends heavily on the number of triangles, since the clustering coefficient is the percentage of triangles out of the number of triplets (three nodes with two edges) [34, 35]. It is known that triangles are often erroneously obtained using pairwise correlations [11]. In the simulations, this problem can be observed in each of the four network topologies (see Fig 4). When using pairwise correlations to determine the connections in the network (red curve), the small-worldness index is much higher than the true value for each of the networks (dashed line), whether they are small-worlds or not. It even appears that, for pairwise correlations, the index increases as the numbers of observations increases. The shrinkage (blue curve) and lasso (green curve) estimates appear to be the most accurate in general. Thus, as expected, due to overestimation of the prevalence of triangles, the pairwise correlation method clearly inflates the clustering coefficient (Fig 5). When considering only pairs of regions, the number of triangles will be high when the correlations in the indirect connection are high [52]. Fig 4 also shows that obtaining too many connections (20%) results in lower estimates of small-worldness, but this is mainly due to the ensuing underestimation of the average pathlength (Fig 5), since the clustering coefficient hardly changes (Fig 5).

### Fragmentation and Connectedness

**Fig 6:**
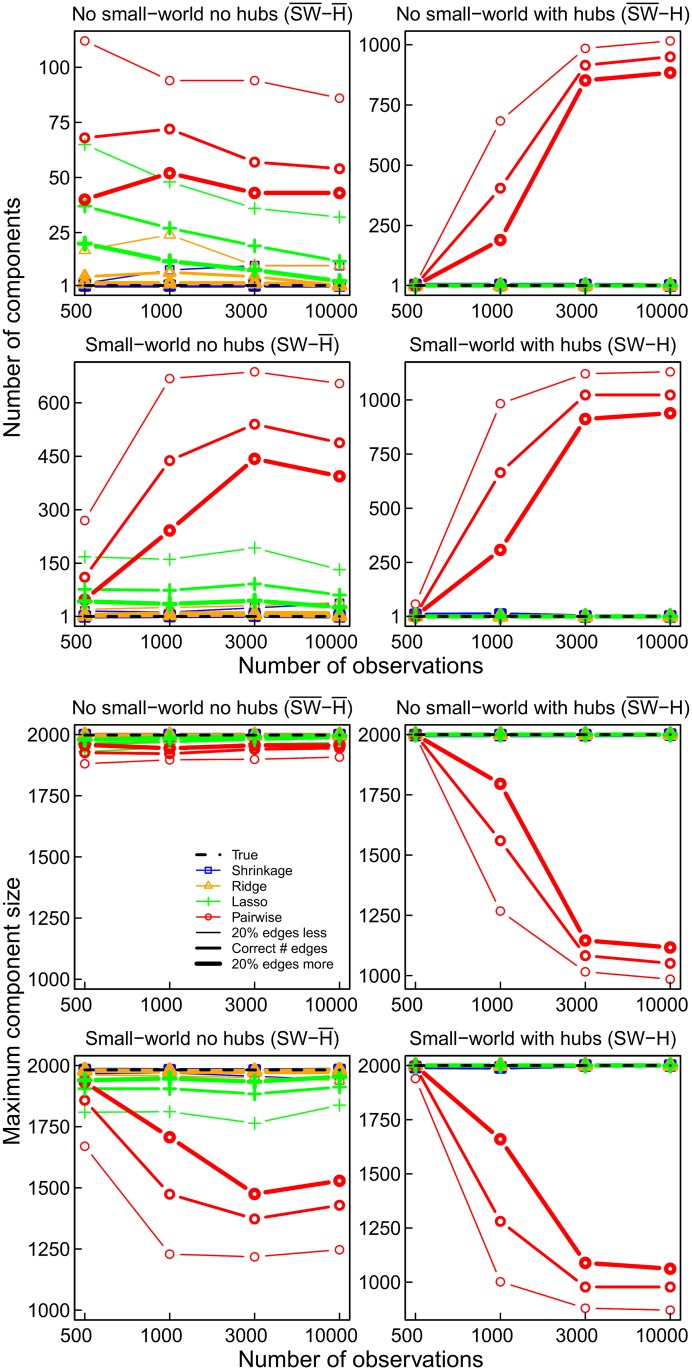
The number of components (upper) and the size of the largest component (lower) obtained for the four networks and estimation methods.

In the true networks each pair of nodes is directly or indirectly connected, which implies that there are no isolated (groups of) nodes. However, a network obtained by using pairwise correlations is fragmented into many smaller ‘islands’, that is, isolated components, up to as many as 1000 in the network with hubs (Fig 6). Of course this is accompanied by components of smaller size. The size of the largest component is smaller up to a factor of 2 than for a component in the partial correlation network (Fig 6). Partial correlation methods, in particular the ridge regression and shrinkage methods, result in less fragmented and actually connected networks.

### Betweenness Centrality

**Fig 7:**
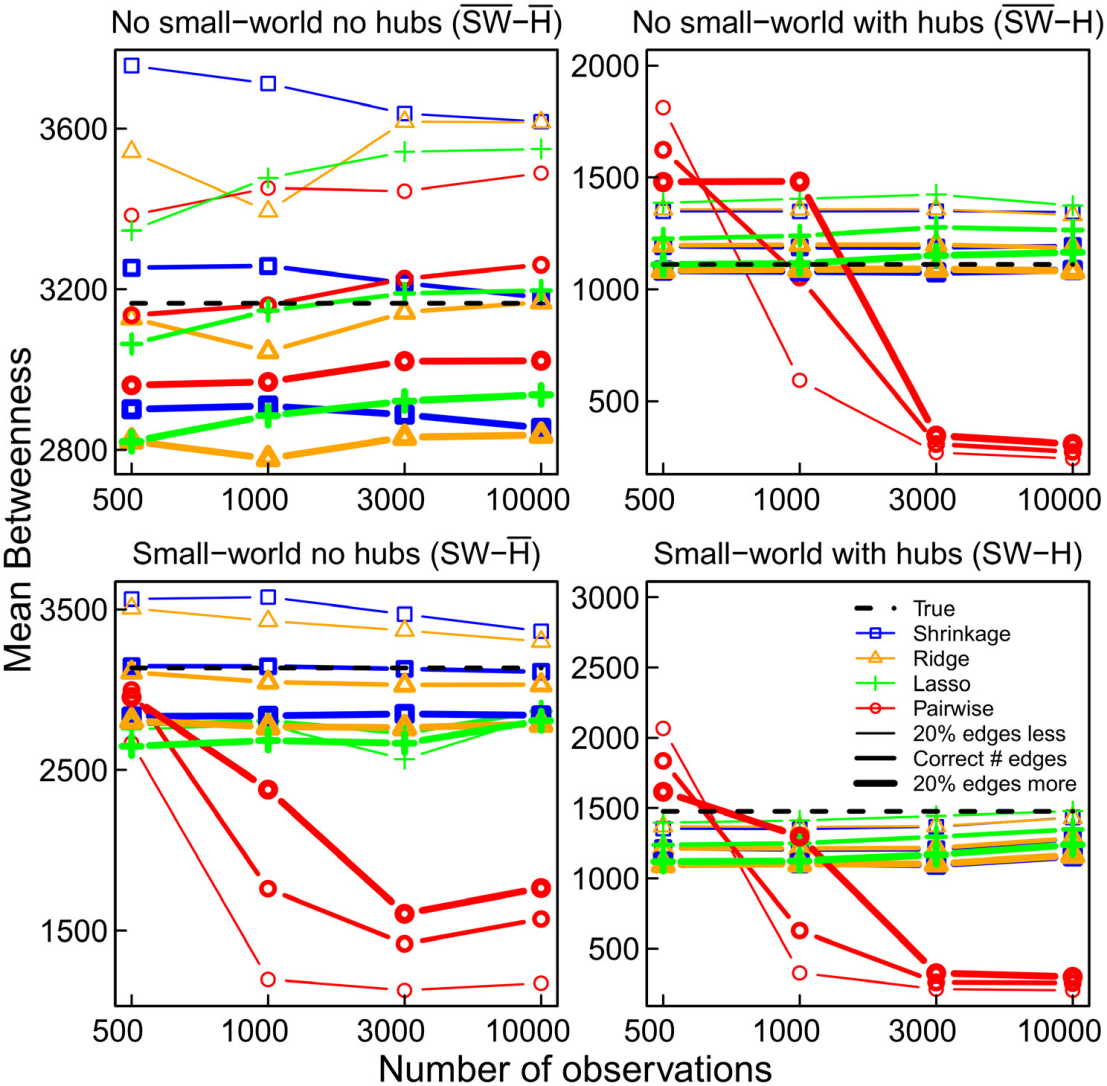
The mean betweenness centrality of the four networks and estimation methods.

The average betweenness centrality of the estimated networks, that is the average of the number of shortest paths on which each node lies, is also affected by the use of pairwise correlations. In particular, in those networks, in which using pairwise correlations resulted in strong fragmentation of the network ($\text{SW}-\bar{\text{H}}$, SW-H, $\bar{\text{SW}}-\text{H}$), the average betweenness centrality is substantially underestimated, as the total number of shortest paths is reduced in the pairwise correlation networks (Fig 7).

### Degree Distribution

As mentioned above, the degree of a node refers to the number of connections it has with other nodes. The degree distribution of a network is important, as it has been connected with properties like preferential attachment (“the rich get richer”; [27]). We investigated whether estimates of the networks in the simulation scenarios provided a good representation of the degree distribution. The true and recovered degree distributions of the four networks are shown in Fig 3. A network obtained with pairwise correlations tends to have too many nodes with low degree, as the mode is too low, whereas most networks obtained with partial correlations are closer to the true distribution (see Fig 3).

**Fig 8:**
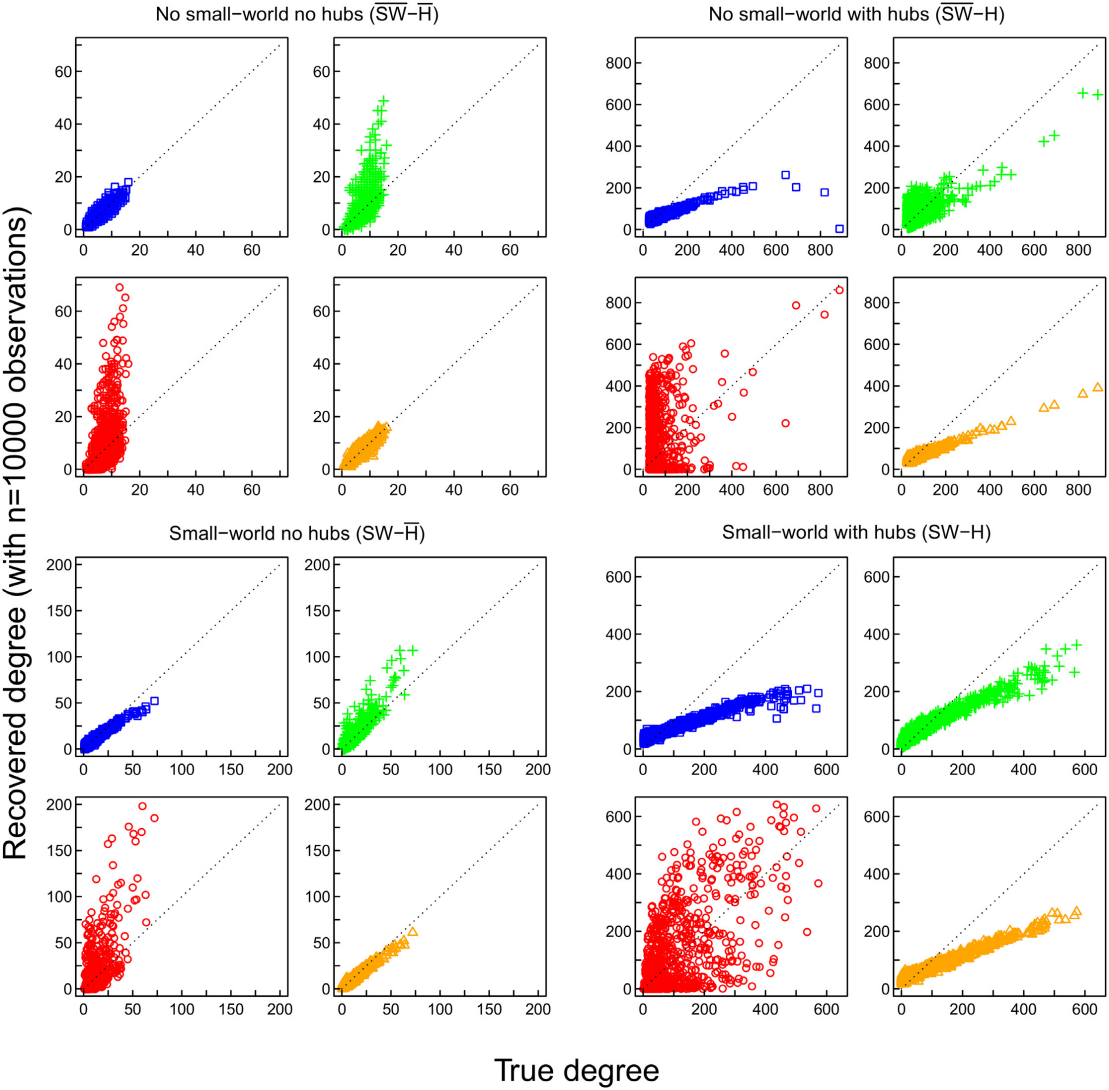
Recovery of node degrees based on 10000 observations. Scatter plots of true (x-axis) vs recovered (y-axis) node degrees of shrinkage (blue), ridge (orange), and lasso (green) estimated partial correlations, and of pairwise correlations (red).

**Fig 9:**
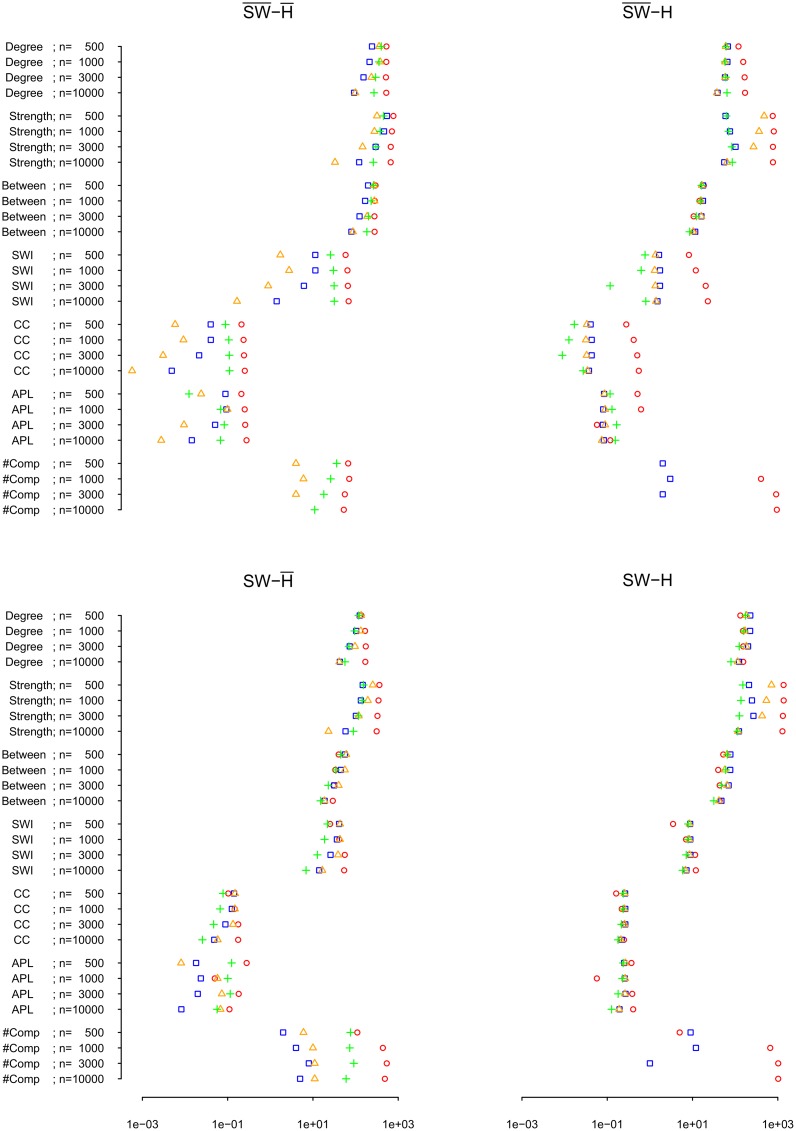
Overview of absolute differences between true and recovered network characteristics of shrinkage (blue), ridge (orange), and lasso (green) estimated partial correlations, and of pairwise correlations (red), in the condition where the correct number of edges is selected. For node characteristics (i.e., degree, strength, and betweenness), sums of absolute differences of linearly transformed variables x* are shown ($x^{*}=\frac{\left(x-Min(truevariable)\right)}{\left(Max(truevariable)-Min(truevariable)\right)}$; i.e., 0 was mapped on the minimum of the *true* variable, and 1 was mapped on the maximum of the *true* value). SWI = Small-worldness index, CC = Clustering coefficient, APL = Average path length, #Comp = Number of components, n = Number of observations. NB: x-axis on logarithmic scale; if absolute difference is zero, the method’s symbol is not shown.

Correctly reproducing the underlying distribution of degrees does not necessarily imply that the nodes with low degrees indeed have low degrees and the nodes with high degrees indeed have high degrees, that is, that the degrees of the individual nodes are reproduced faithfully. Therefore, we compared the recovered degrees of the nodes to their true degrees. This comparison showed that pairwise correlation networks have a tendency to contain several nodes with much higher degree than the true network (Fig 8). In contrast, the partial correlation networks tend to underestimate the true degrees, but in general are closer to the degree distribution than the pairwise correlation network. Furthermore, the misfit between recovered and true degrees decreases for the partial correlation networks with longer time-series, but not so for the pairwise correlation networks (Fig 9). Weighted degrees (strengths) of the network nodes were in all conditions better estimated by partial correlation methods than by pairwise correlation (Fig 9).

### Summary of Network Characteristics

The previous sections addressed in detail the method’s biases, including over- or underestimation, in the recovery of network characteristics at different edge selection criteria. Summarizing, Fig 9 shows an overview of the absolute differences between true and recovered network characteristics of the four networks. Overall, partial correlation methods tend to be closer to the true network characteristics, that is, the recovered network is more representative of the true network with respect to the network characteristics than the network recovered by pairwise correlations. Furthermore, partial correlation methods in most cases improve with increasing time-series length, while this is not the case for pairwise correlations. Naturally, even if a recovered network has similar network characteristics as the true network, this does not imply that the recovered connections between nodes represent true connections in the network, which is addressed in the next section.

### Correct Connections

**Fig 10:**
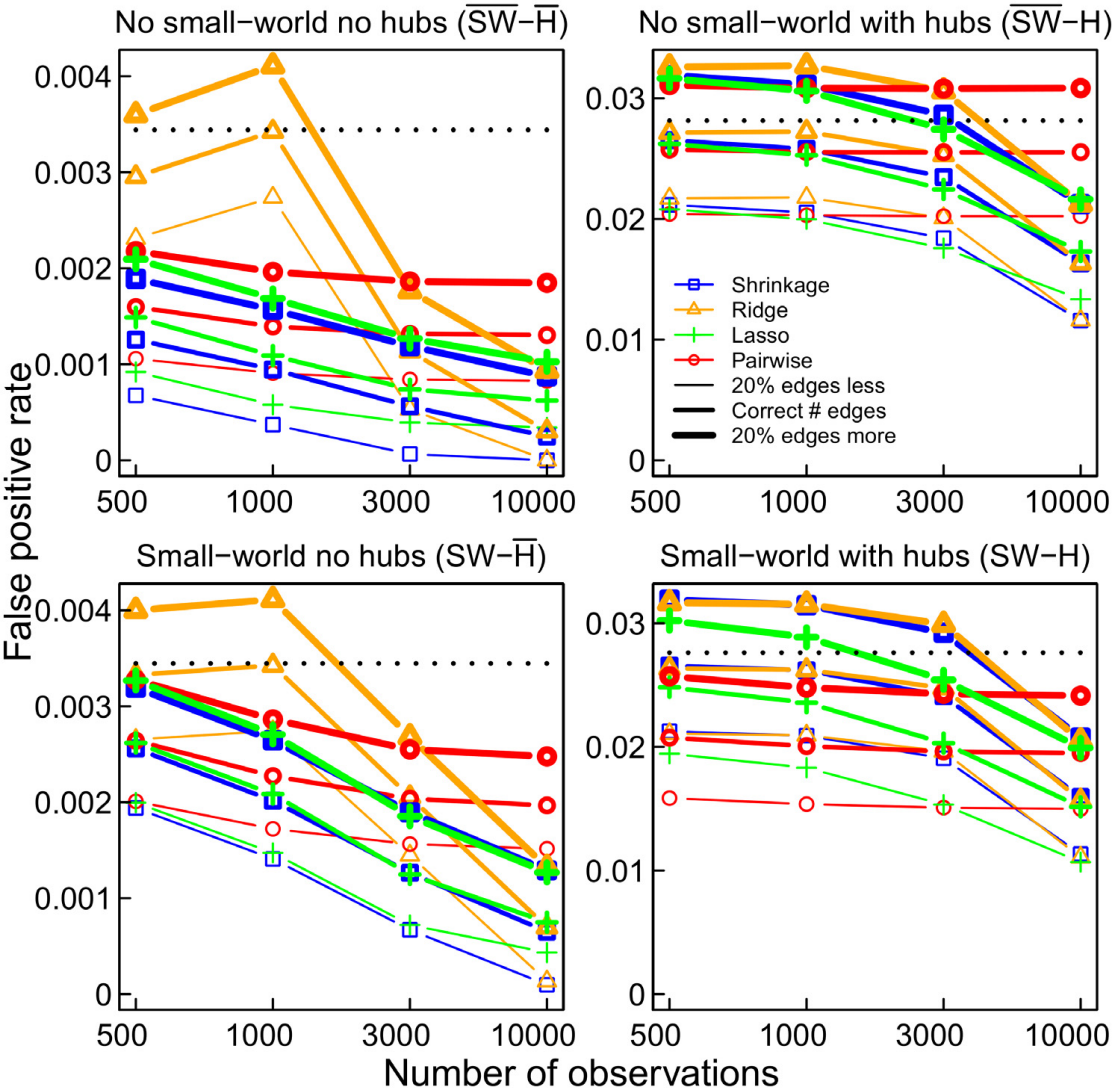
The false positive rate for the four networks and estimation methods. The dotted line ⋯ shows the level of the false positive rate above which the absolute number of false positive edges even exceeds the absolute number of edges in the true network.

To consider to what extent connections were correctly identified, we examine the false positive rate (FPR), that is, the probability of deciding that there is a connection given that there is no true connection, and the true positive rate (TPR), that is, the probability of deciding that there is a connection given that there actually is one. The FPRs of the methods, shown in Fig 10, may seem small considering their absolute values. However, as the networks were sparse, the number of erroneously inferred edges is divided by a very large number of non-existent connections. In order to set FPRs into perspective, the proportion of edges in the true network is indicated as well (dotted line). The FPR of the pairwise correlation networks is nearly always higher than that of the lasso and shrinkage based partial correlation networks (Fig 10). Ridge regression partial correlation networks have an unacceptably large FPR if the number of observations is smaller than the number of nodes, as expected. In most cases, the FPR is lower than the proportion of edges in the true network (dotted line). However, this result does not occur in the presence of hubs.

**Fig 11:**
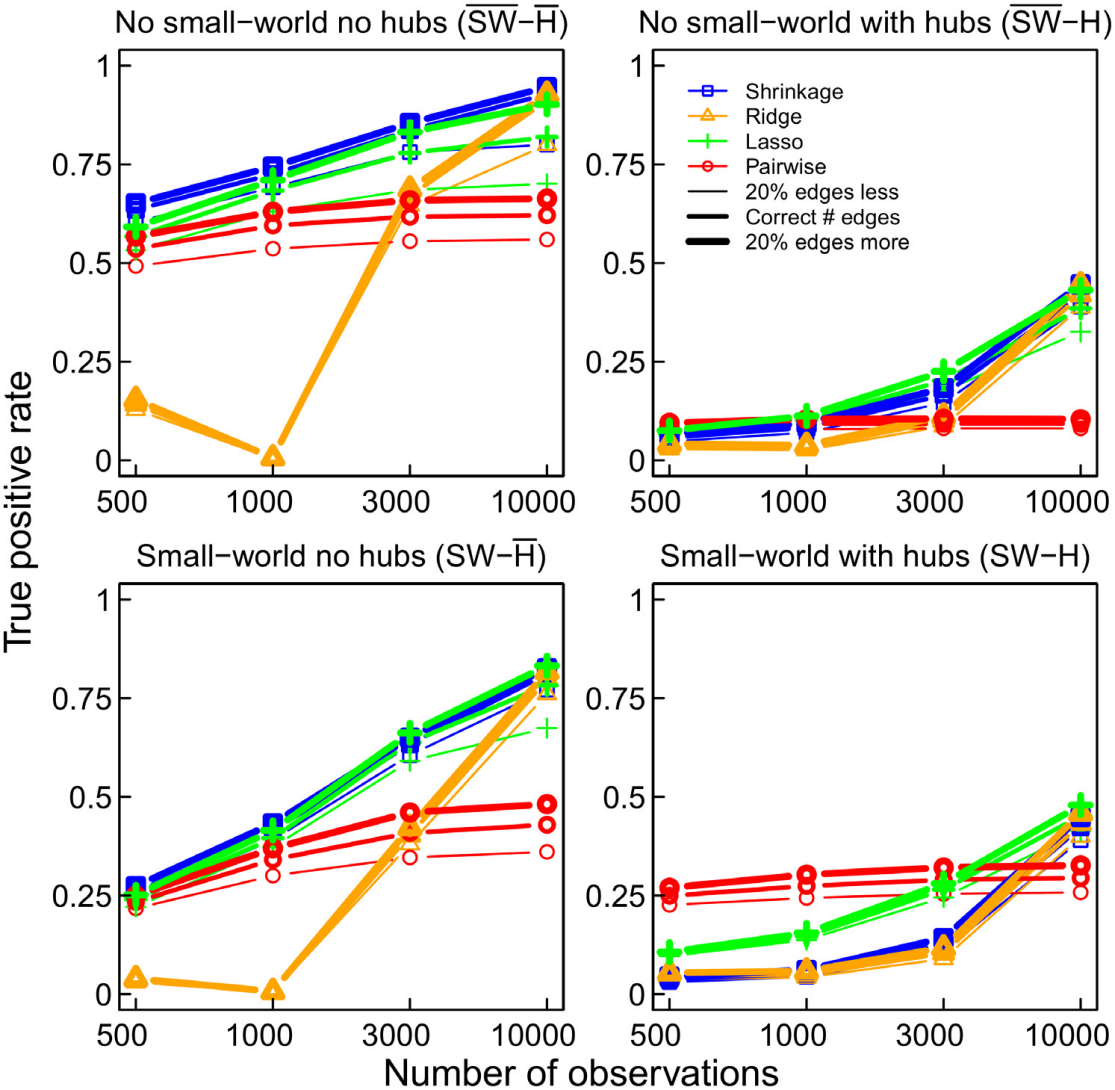
The true positive rate for the four networks and estimation methods.

**Fig 12:**
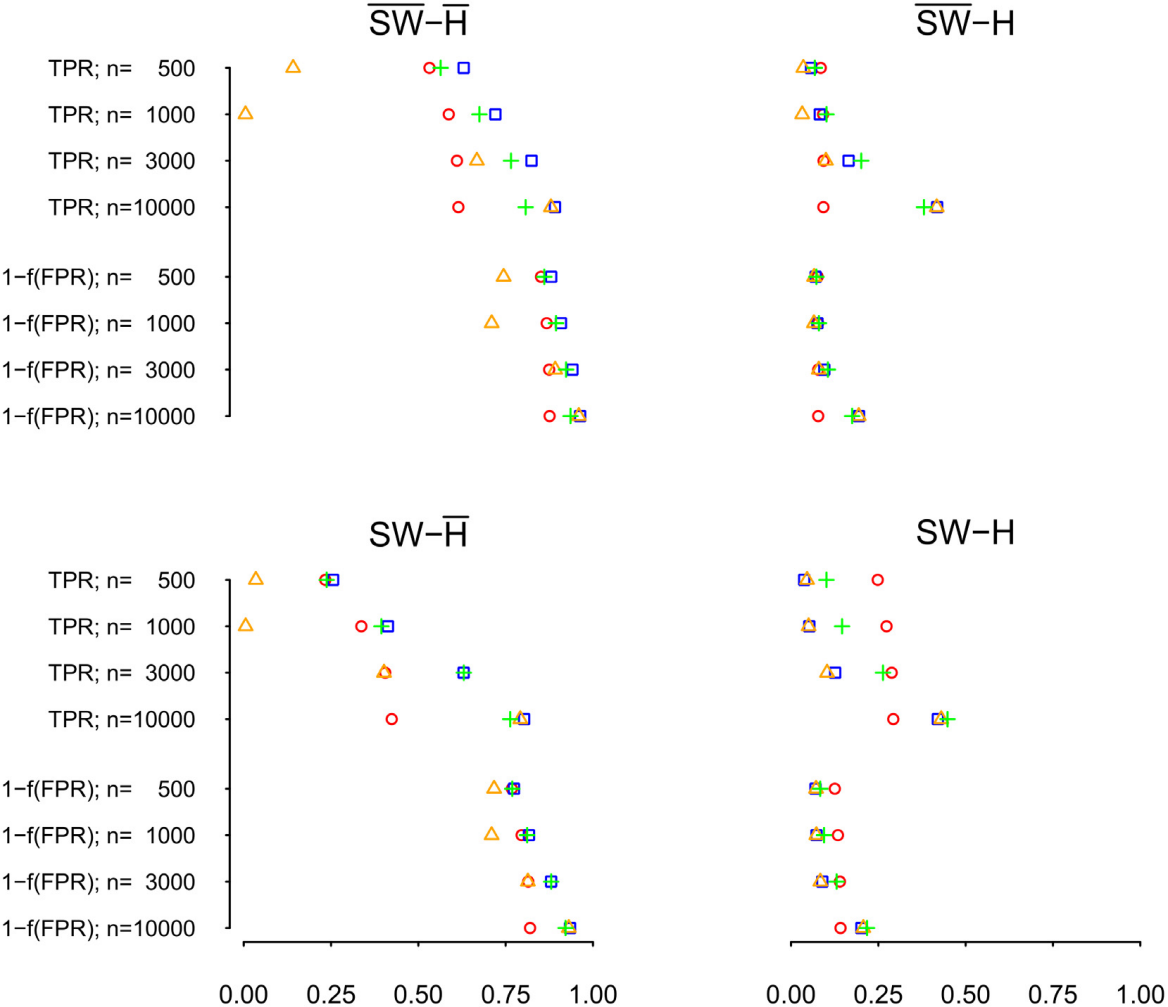
Overview of TPR and of 1 − *f*(FPR) for shrinkage (blue), ridge (orange), and lasso (green) estimated partial correlations, and for pairwise correlations (red), averaged over all three selection criteria (i.e., correct number of edges, 20% less edges, and 20% more edges). *f*(FPR) = exp(−10^2^*FPR); *n* = Number of observations.

The TPR in Fig 11 also shows that pairwise correlation networks are inaccurate in most cases, and that ridge regression partial correlation networks estimated from small numbers of observations are inaccurate. Strikingly, the pairwise correlation networks show almost no improvement with increasing numbers of observations. This indicates that pairwise correlation networks are in general inappropriate for inferring underlying connectivity. In contrast, ridge regression partial correlation networks do improve with increasing numbers of observations, reaching TPR and FPR values comparable to lasso and shrinkage based networks with 10000 observations. Note that the TPR is not particularly high for any type of method; however, for partial correlation networks it increases strongly with increasing number of observations. Recall that with 2000 nodes a total of nearly two million possible edges are estimated with 10000 observations, which is a poor ratio of observations to possible edges (parameters). To summarize, Fig 12 gives an overview of TPRs, and a function of FPRs (such that a higher value is associated with a better FPR) for the four methods and the four networks topologies. While partial correlation methods reach average TPRs larger than.75 in the two networks without hubs with sufficient numbers of observations, the average TPR in the two hub networks remains very low (< .5) for all methods at the numbers of observations considered in our simulations.

**Fig 13:**
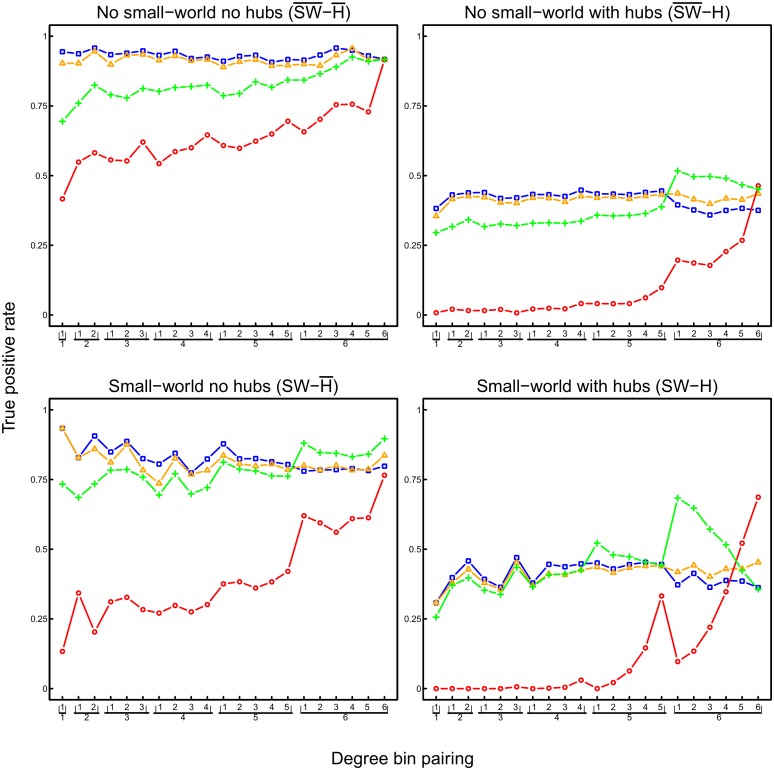
True positive rate as a function of node degree (given 10000 observations) of shrinkage (blue), ridge (orange), and lasso (green) estimated partial correlations, and of pairwise correlations (red). For each network, nodes were divided into 6 bins according to degree: 5 equally-sized bins, and a 6th bin containing the 50 nodes with the highest degree (i.e., the hubs in the hub networks). TPR is shown for each pairing of degree bins (e.g., sixteenth pair $\left(\frac{1}{6}\right)$ refers to edges between the nodes with lowest degrees and the nodes with highest degrees; rightmost pair $\left(\frac{6}{6}\right)$ refers to edges between the nodes with highest degrees).

**Fig 14:**
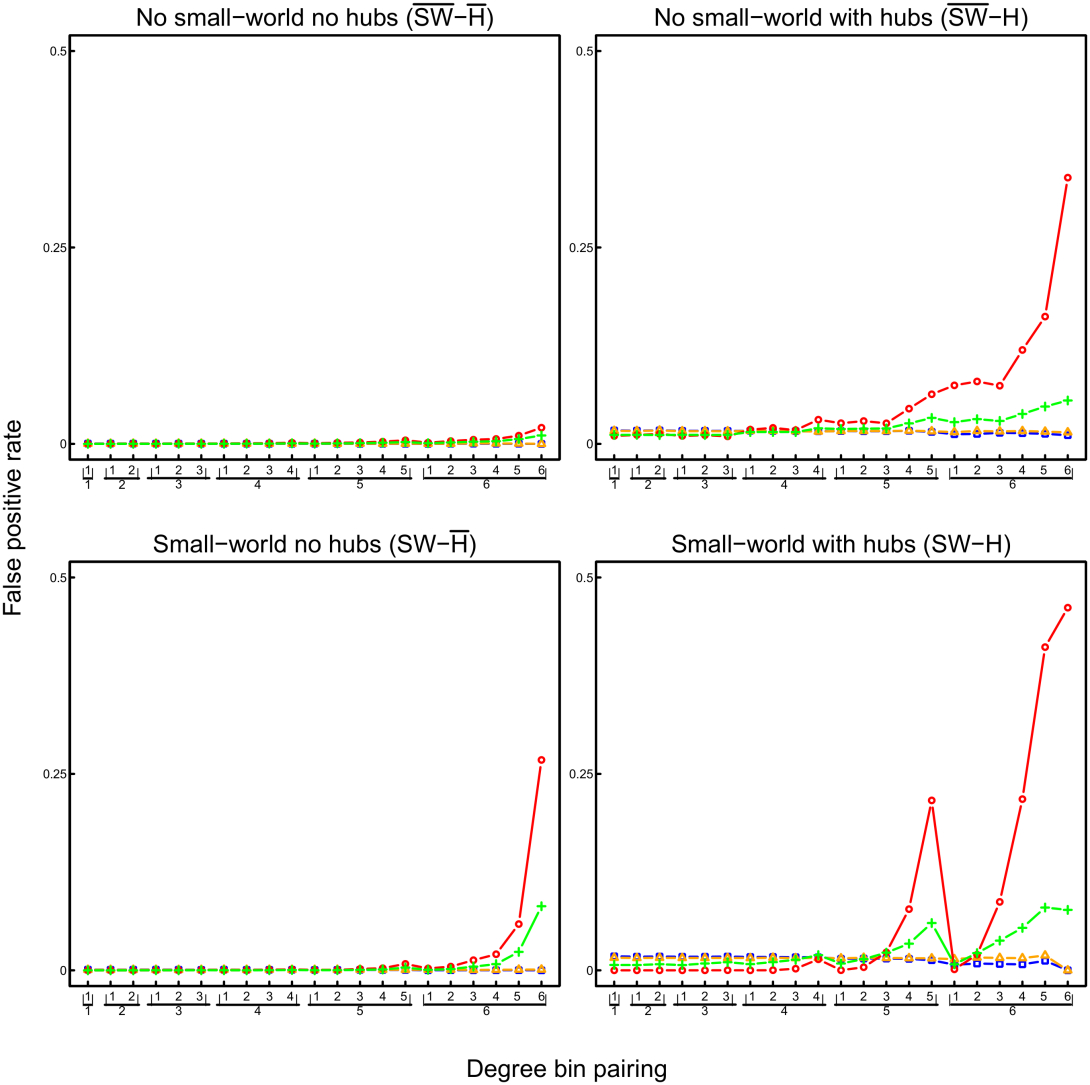
False positive rate as a function of node degree (given 10000 observations) of shrinkage (blue), ridge (orange), and lasso (green) estimated partial correlations, and of pairwise correlations (red). For each network, nodes were divided into 6 bins according to degree: 5 equally-sized bins, and a 6th bin containing the 50 nodes with the highest degree (i.e., the hubs in the hub networks). FPR is shown for each pairing of degree bins (e.g., eleventh pair (1,6) refers to edges between the nodes with lowest degrees and the nodes with highest degrees; rightmost pair (6,6) refers to edges between the nodes with highest degrees).

We also examined whether the identification of a true connection depends on the degrees of the two nodes that are connected by it (e.g., are connections between nodes with two degrees more easily identified than connections between a hub node and a node with two degrees?). For this purpose, we calculated the TPR and the FPR as a function of the true degrees of each pair of connected nodes. Fig 13 shows that the TPR is higher in the partial correlation networks than in the pairwise correlation networks for almost all degree pairings. Pairwise correlation networks have a very low TPR for connections between lowest to larger degree nodes. Merely for connections involving largest and hub nodes does the TPR of pairwise correlation networks approach or exceed the TPRs of the partial correlation networks. However, in exactly these cases, the FPR of the pairwise correlation networks are inacceptably large (Fig 14). The graphical lasso networks have somewhat elevated FPRs and TPRs for connections between hub nodes. In contrast, the FPR of the other two partial correlation networks remains relatively small across low, medium, large degree and hub nodes, while their TPRs are in general the highest (> .75 for networks without hubs, and ranging between .25 and .5 for networks with hubs) and relatively stable across the whole range of lowest degree to hub nodes.

### Effect of Hubs

**Fig 15:**
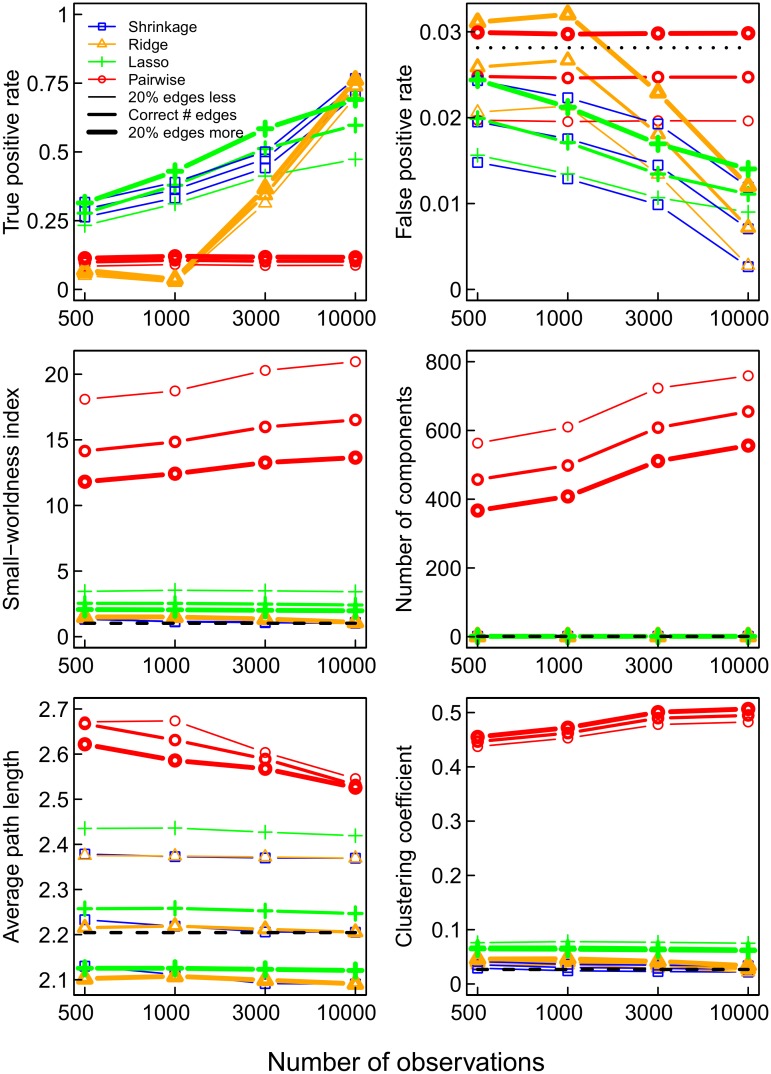
Recovery results of the four estimation methods for additional random network $\bar{\text{SW}}-\bar{\text{H}}-\text{c}$ (with the high density of 3%). True −− network metrics indicated where appropriate. The dotted line ⋯ shows the level of the false positive rate, above which the absolute number of false positive edges even exceeds the absolute number of edges in the true network.

As shown above, in the two networks with hubs, all methods perform worse. In these networks, the maximum degree is much larger than in the networks without hubs (see Table 1). However, these two networks also have a larger number of edges (density of 3%), in order to make a network with large-degree nodes and still be connected, than the networks without hubs (density of 0.3%). To separate the effects of density and hubs, we analyzed the complementary random network (i.e., without hubs) with a density of 3% ($\bar{\text{SW}}-\bar{\text{H}}-\text{c}$). The results support the hypothesis that the presence of hubs causes the decrease in perfomance, rather than the lower density of the network. The true positive and false positive rates of network $\bar{\text{SW}}-\bar{\text{H}}-\text{c}$ (Fig 15) show much better performance of the partial correlation networks than the pairwise correlation networks with hubs ($\bar{\text{SW}}-\text{H}$ and SW-H, Figs 10 and 11), but also, slightly worse performance than in the sparser random network $\bar{\text{SW}}-\bar{\text{H}}$.

In all cases, pairwise correlations perform badly, as in the sparser random network $\bar{\text{SW}}-\bar{\text{H}}$. For this reason, and on the basis of the recovery of the other characteristics of network $\bar{\text{SW}}-\bar{\text{H}}-\text{c}$ by the four methods (Fig 15), the large difference in recovery between networks $\bar{\text{SW}}-\bar{\text{H}}$ and $\text{SW}-\bar{\text{H}}$ vs SW-H and $\bar{\text{SW}}-\text{H}$ can indeed be attributed to the presence of hubs.

## Results of Application to Resting-State Data

To illustrate how these results affect the analysis of actual neuroimaging data, we applied pairwise and partial correlation methods to time series of BOLD resting-state data, obtained from 5 individuals, similar in terms of genetic makeup. Resting-state functional connectivity maps were constructed through the hierarchical decomposition of the cortical surface into 5 embedded cortical parcellations with number of ROIs (nodes) *n* of 68, 114, 219, 448, and 1000 [36, 46, 48, 49]. To compare the methods, the resting-state time series obtained from each parcellation was analyzed with both pairwise correlations and partial correlations. We chose to obtain partial correlations by optimal shrinkage estimation, as it was in our simulations in general preferrable above ridge regression, and although quite similar to the lasso, seemed slightly better than the lasso, as judged by the TPRs. For each participant, we calculated pairwise correlation and partial correlation networks consisting of the 3% strongest (pairwise or partial) correlations for each of the parcellations (resulting in 68, 193, 716, 3003, and 14985 edges, respectively). We focus on three issues: a) the difference between correlation and partial correlation networks, b) the consistency of the networks with respect to different parcellations (i.e., with the increasing number of ROIs), and c) the consistency of the estimated networks with varying numbers of observations (i.e., lengths of the time series).

### Pairwise Correlation vs Partial Correlation Networks

**Fig 16:**
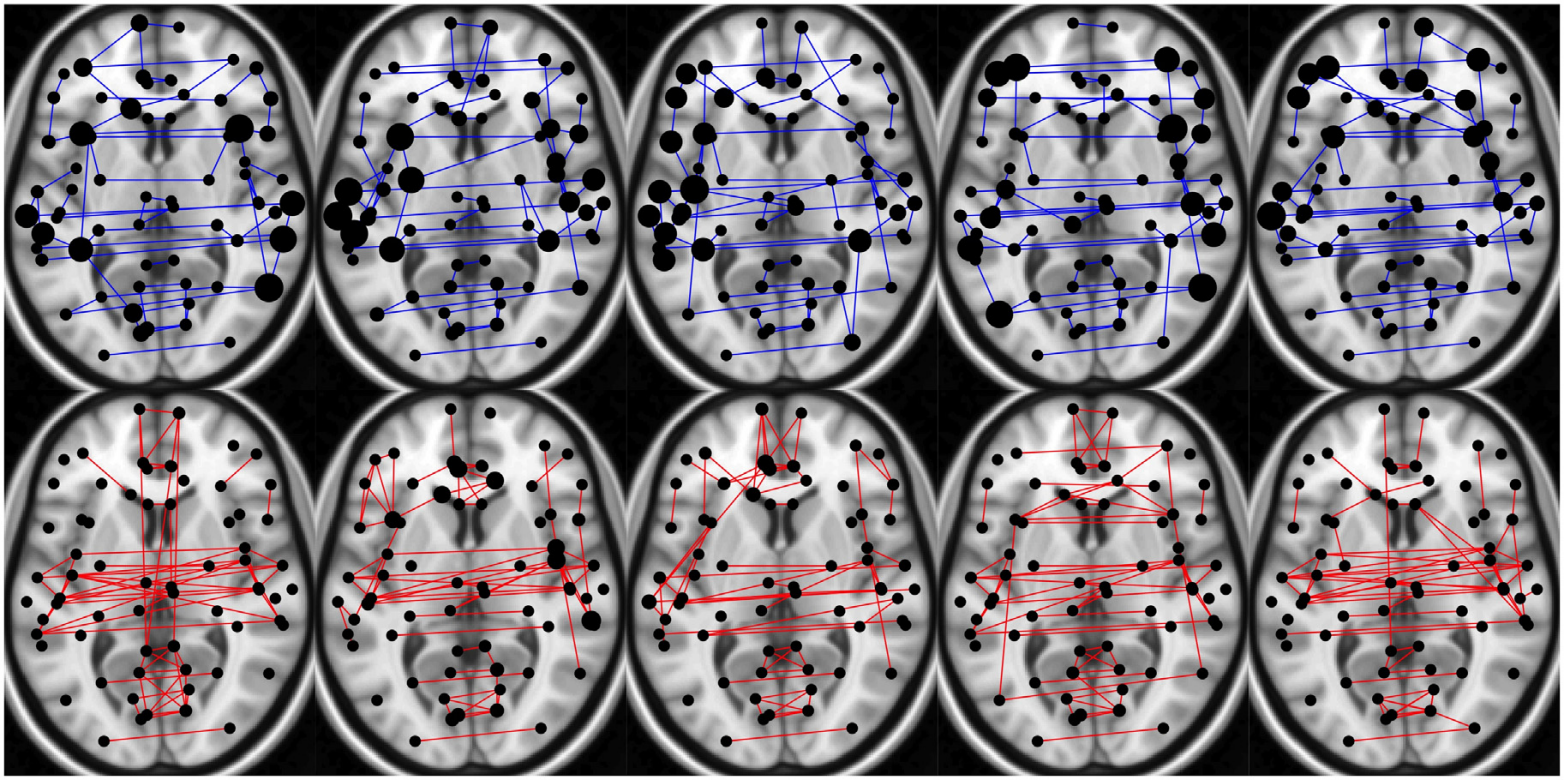
Networks of 68 ROIs based on 3% strongest partial correlations (blue) and pairwise correlations (red) of all 5 participants. Left hemisphere is on left side. ROIs with larger nodes have higher betweenness centralities. Networks are superimposed on transverse MNI152 T1 template for illustration purposes (Copyright (C) 1993–2004 Louis Collins, McConnell Brain Imaging Centre, Montreal Neurological Institute, McGill University). Figure prepared with the R-package *qgraph*.

**Fig 17:**
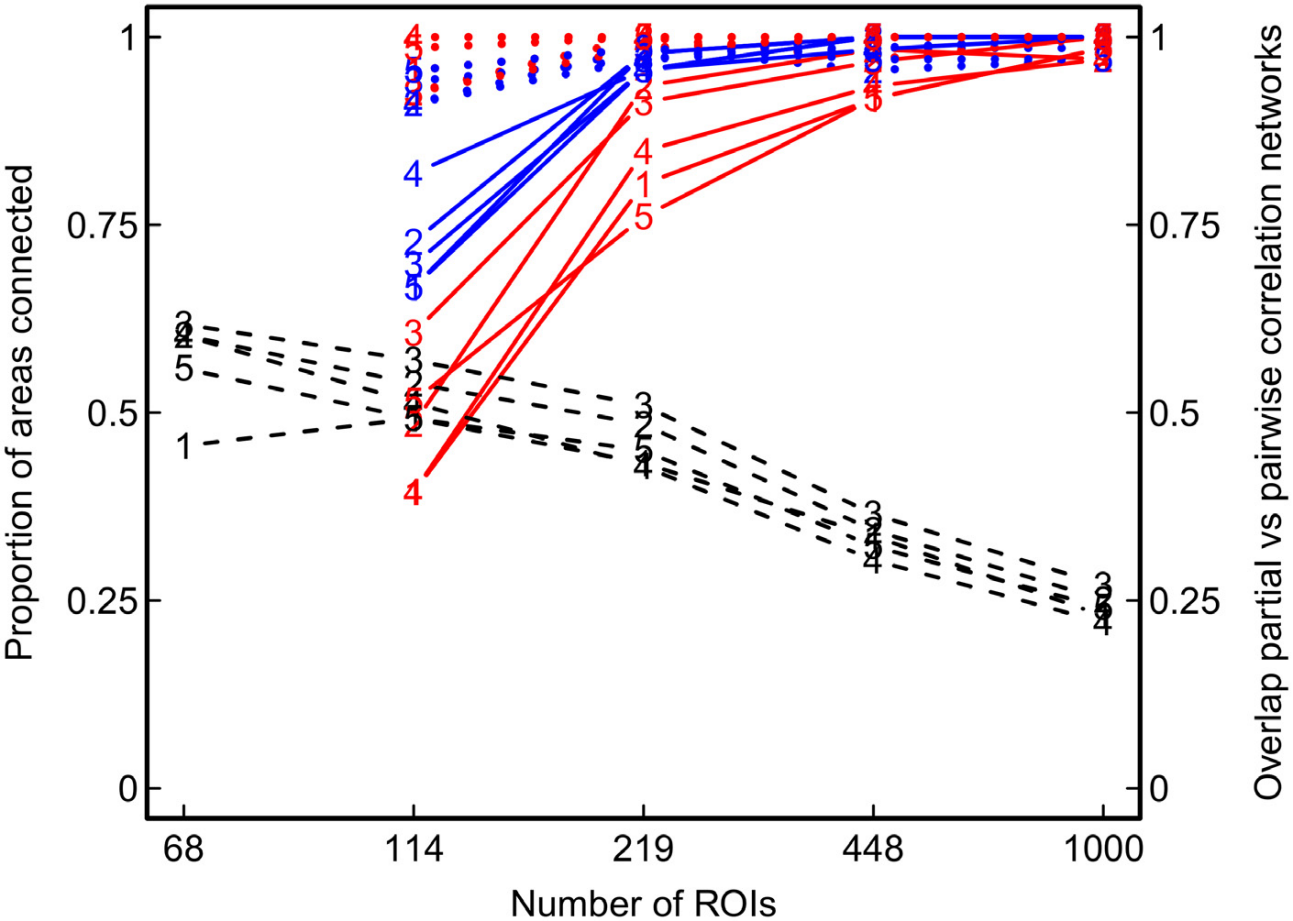
Overlap between networks at different numbers of ROIss (parcellations). Dashed black lines −− show the proportion of edges that were present both in the pairwise and in the partial correlation network of a given parcellation. Separate lines for each participant (numbered 1–5). Blue (or red) lines show the comparison of the base-line 68-ROI parcellation with higher-resolution parcellations for pairwise correlation (red) networks (or partial correlation (blue) networks). Plain blue (or red) lines − show the proportion of areas of low-resolution parcellation that were internally connected by at least one edge in the higher-resolution parcellations, given that the area was split (within-area connectivity). Dotted blue (or red) lines … show the proportion of areas that were inter-connected in the low-resolution parcellation, that were also inter-connected by at least one edge in the higher-resolution parcellations (between-area connectivity).

**Fig 18:**
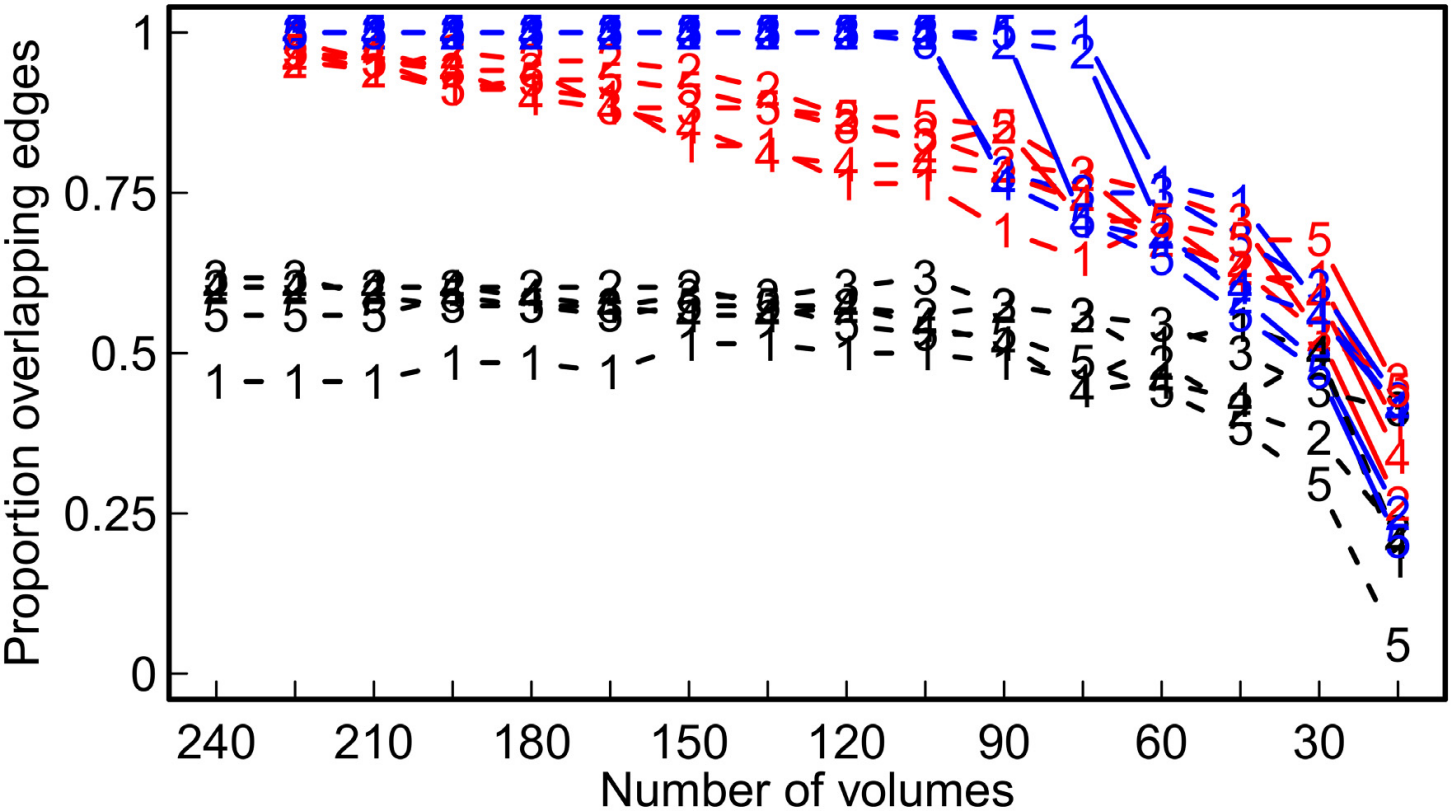
Overlap between networks at different numbers of volumes (i.e., time-series lengths). Shown is the proportion of identical edges present in two respective networks. Black lines −− show overlap between the pairwise correlation network and the partial correlation network of a participant, based on a given number of volumes (i.e., time-series length). Separate lines for each participant (numbered 1 − 5). Red (or blue) lines indicate overlap between the pairwise correlation (red) (or partial correlation (blue)) network based on the full time-series of 240 volumes and the pairwise correlation (red) (or partial correlation (blue)) network based on smaller numbers of volumes (i.e., shorter time-series length.

**Fig 19:**
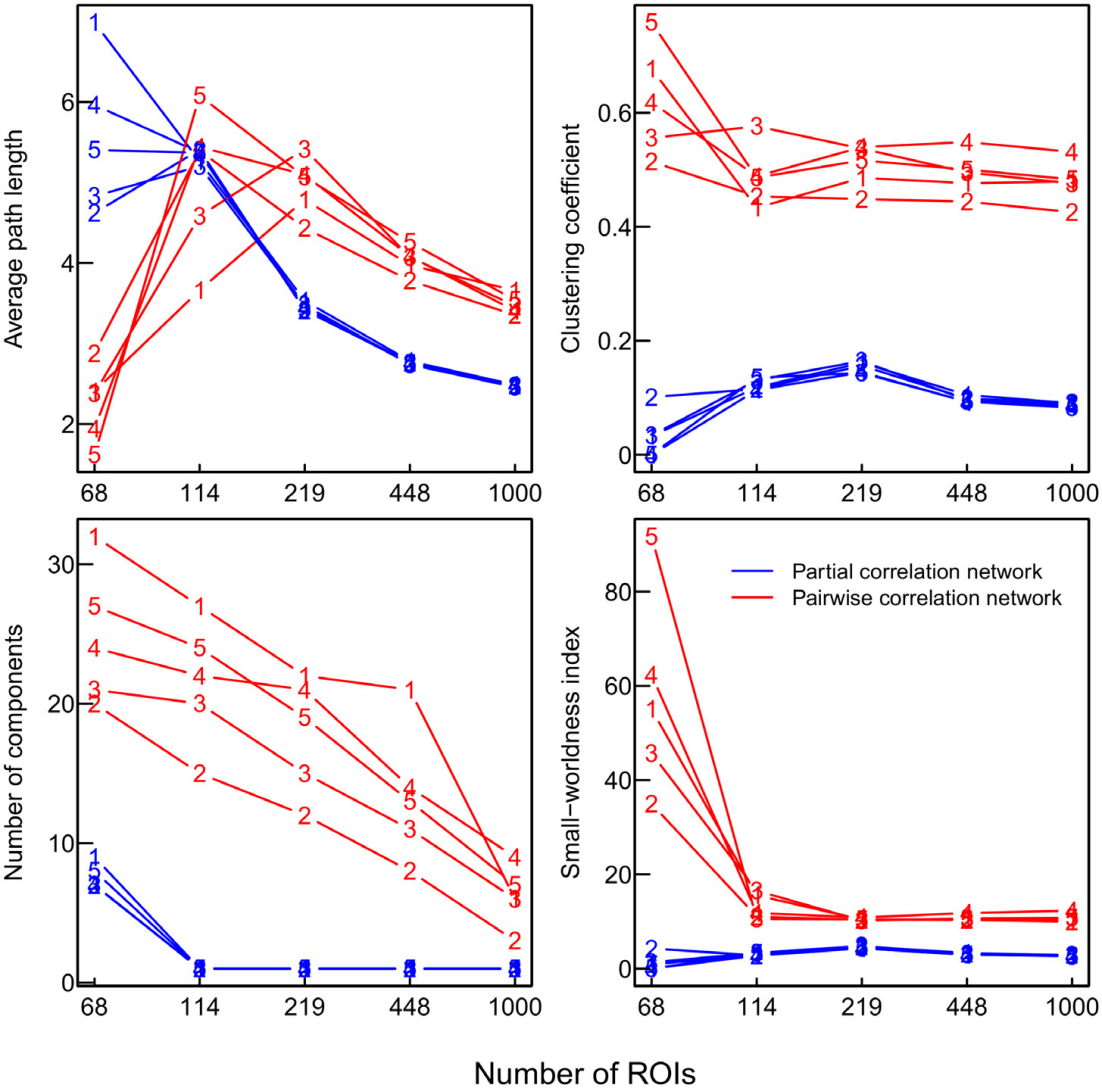
Global network metrics of interest of pairwise correlation (red) and partial correlation (blue) networks for different numbers of ROIs (parcellations). Numbered lines for participants 1 to 5.

Fig 16 shows the obtained networks of the 3% strongest partial or pairwise correlations in the five participants. Both in the pairwise and in the partial correlation networks of all participants, those areas commonly reported as associated with resting-state activity (i.e., we considered precuneus, medialfrontal, inferior parietal, medial temporal lobe, primary sensorimotor, primary visual, extrastriate visual, bilateral temporal, insular, anterior cingulate cortex, superior parietal, superior frontal, posterior cingulate cortex, in line with [53–57]) had a larger average degree and a larger average betweenness than the remaining areas. However, the amount of overlap between pairwise and partial correlation networks was 62% at most, and decreased further with increasing number of ROIs or decreasing number of observations in each participant (see dashed black lines in Figs 17 and 18, respectively). As expected, network characteristics that depend on the inferred network topology differ substantially depending on the method used. Fig 19 shows network metrics of interest for the five participants over different parcellations and methods. As in the simulation study, the use of pairwise correlations results in more fragmented networks with a higher amount of clustering and a higher small-worldness index.

**Fig 20:**
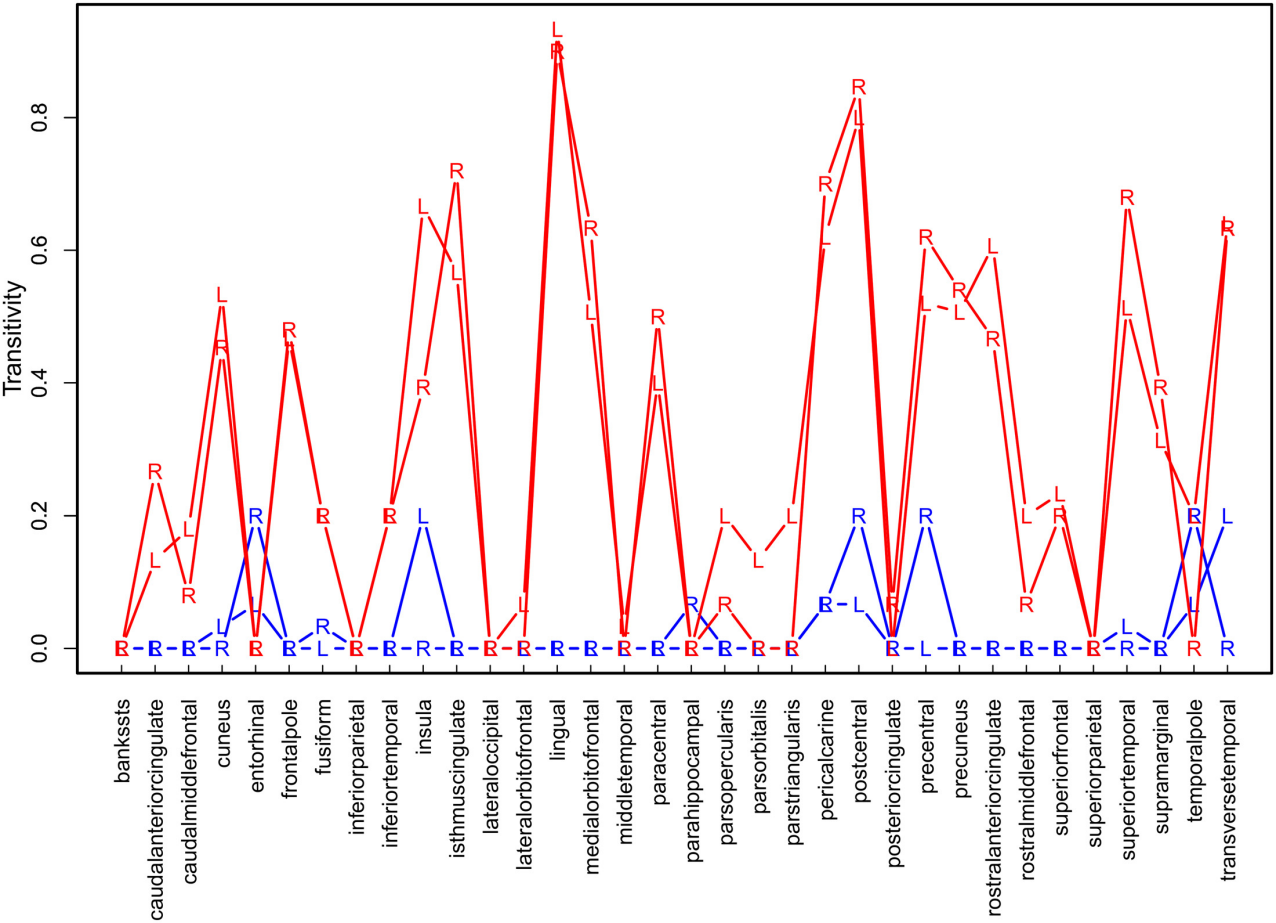
Local transitivity of left (L) and right (R) hemisphere ROIs in pairwise correlation (red) and partial correlation (blue) networks with 68 ROIs, averaged over participants.

As shown in Fig 20, the local transitivity of most ROIs is larger in the pairwise correlation network than in the partial correlation network. This is in line with the expectations based on theory and our simulation results.

**Fig 21:**
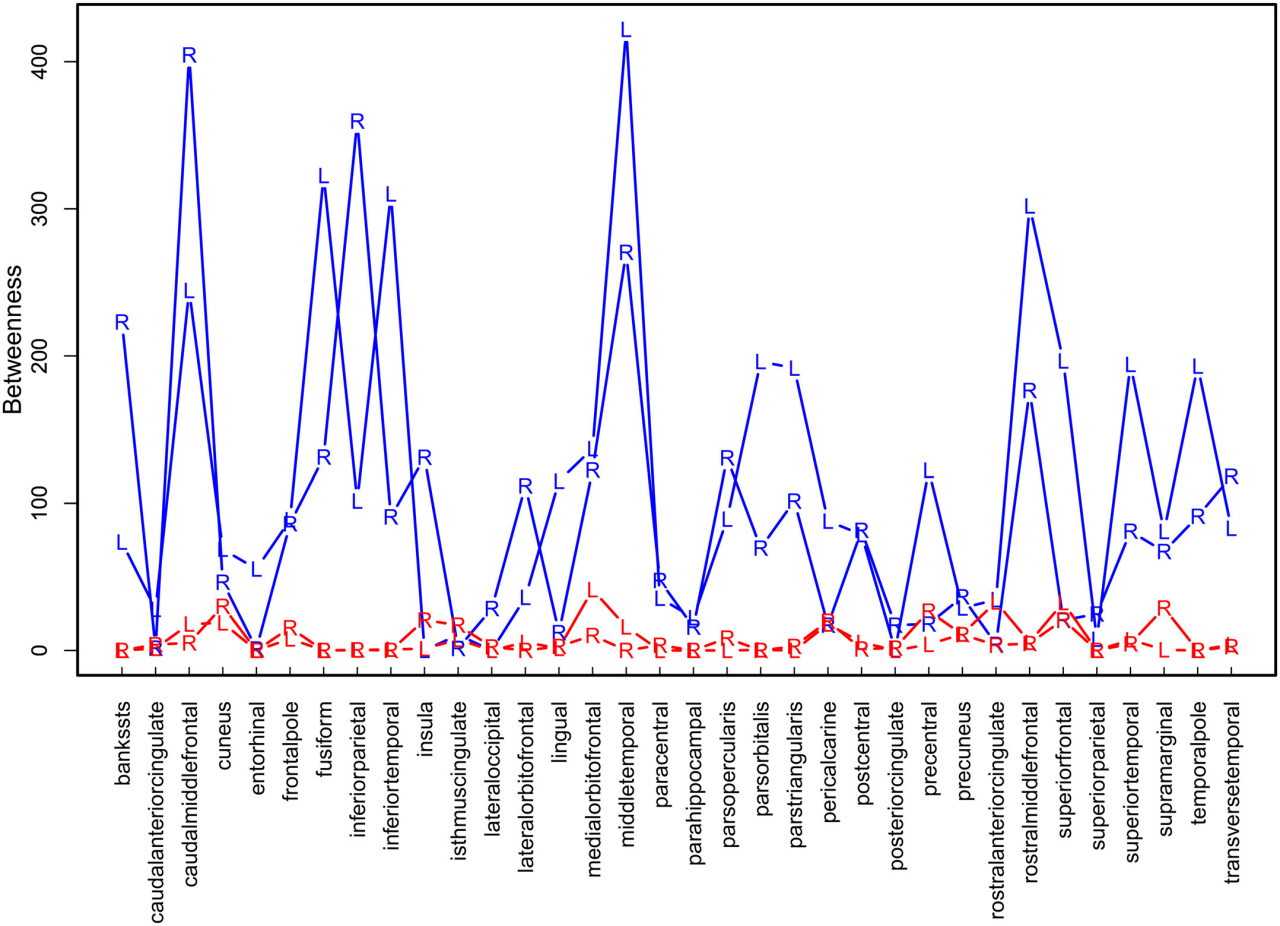
Betweenness centrality of left (L) and right (R) hemisphere ROIs in pairwise correlation (red) and partial correlation (blue) networks with 68 ROIs, averaged over participants.

Betweenness centralities of each ROI are shown in Fig 21. In line with our simulation results, in which pairwise correlation networks resulted in a severe underestimation of mean betweenness centrality if the number of observations was sufficiently large, the average betweenness centrality of the pairwise correlation networks (red line) is much smaller than the average betweenness centrality of the partial correlation networks. This is the case for almost all ROIs.

### Network Consistency Across Different Parcellations

To examine the overlap between networks of low-resolution and higher-resolution parcellations, we focussed on within-area connectivity and between-area connectivity (Fig 17). Between-area connectivity (given a connection in the 68 ROI parcellation) is high in pairwise and in partial correlations networks. However, within-area connectivity is higher in partial correlation networks than in pairwise correlation networks.

### Network Consistency Across Varying Time-Series Lengths

From each participant, we prepared 16 embedded data-sets with consecutively shorter length of the time-series, starting with the full series of 240 volumes down to a minimum of 15 volumes. For each data set, we calculated two networks as above, consisting of the edges with the 3% strongest (pairwise or partial) correlations. To assess the overlap of a partial (or pairwise) correlation network based on a given number of volumes with the respective partial (or pairwise) reference network based on 240 volumes, we calculated the proportion of overlapping edges. The proportion of overlapping edges was calculated as the number of individual edges that are present in both networks (i.e., the size of the intersection of the edges in the two networks) divided by the total number of edges in a network (i.e., the 3% of all possible edges that were selected). An overlap of 100% implies that exactly the same edges are present in the two networks, while an overlap of 0% implies that completely different edges are present in the two networks. Partial correlation networks show a 100% overlap between the 240 volumes and consecutively smaller numbers of volumes, down to 90 or 60 volumes (see blue lines in Fig 18). With fewer observations, the overlap decreases. The amount of overlap of the pairwise correlation networks at different time-series lengths is in general lower than or equal to the overlap of the partial correlation networks at different time-series lengths (see red lines below blue lines in Fig 18).

## Discussion

The current study clearly shows that pairwise correlations should not be used to estimate connectivity from functional MRI data, because pairwise correlation networks are generally very poor representations of the true network. Ad-hoc solutions, like tweaking the cutoff threshold for the correlation coefficients, is not a solution because the problem is inherent in the pairwise correlation methodology itself. Pairwise correlations are problematic, because they cannot distinguish between direct and indirect connections, and overestimate the proportion of triangles. We showed that this methodology always results in a small-world network with more components than in the true network, regardless of the true network topology. Additionally, the degree distribution is poorly represented. Logically, in order to correctly infer such network characteristics, a high true positive rate (TPR) and a low false positive rate (FPR) in edge detection are crucial. However, in pairwise correlation networks the TPR is low and does not increase with additional observations (longer time-series), and the FPR of the pairwise correlation networks is nearly always higher than that of the lasso and shrinkage based partial correlation networks.

Small-worldness, degree distribution, betweenness centrality, and number of components are better estimated using the shrinkage or lasso method to obtain partial correlations for large-scale networks. The presence of hubs limited the efficiency of these methods. This is caused by several factors. First, the presence of hubs means that variance explained by a hub node will eliminate other, small signal connections, which leads to lower TPRs. Second, in a network with hubs, the number of small signal connections is relatively large. The reason is that the network (partial covariance matrix) has to represent a proper (non degenerate) distribution, which requires many small signal connections when hubs are present. And the third and final reason is that the maximum number of observations we used is still relatively low compared to the number of parameters (0.005 observations per possible edge, or parameter) [58]. These conditions resulted in the rather poor TPRs for the recovery methods when hubs were present. Thus, the higher the maximum degree in the network, the more independent observations are needed. Naturally, if the sample size is too small, all methods fail. Based on our simulations, we caution against the derivation of brain networks of size 2000 with 500 or less observations. With 500 observations, the TPR of the best methods in a random network is below.75, which is not particularly high. TPR drops dramatically to.25 or below if the network has a more complex structure (small-world networks, and/or networks contains hubs). In this case, clearly, more observations are needed to reasonably infer underlying networks of this size. If obtaining more observations is not possible, networks of smaller size should be considered (i.e., working with less fine-grained parcellations). It should be kept in mind that the simulated datasets contained temporal dependence, as is common in fMRI data and other time-series. As mentioned above, the effective number of observations was thus lower than the actual number of observations [38]. It may be beneficial to use kernel covariance estimators, which are shown to be consistent for time dependent data [59].

While [11] concluded that pairwise correlation can and should be used to measure connectivity in combination with adapted null models, our simulation results suggest otherwise for large-scale networks. The true positive rate and false positive rate of pairwise correlation networks are not acceptable. This also holds for ridge regression partial correlations, but only if sample sizes are smaller than the number of nodes.

In an early simulation study focusing on the recovery of small-world networks with sparse multivariate autoregression (≤ 100 nodes) ridge regression was found to be optimal, with no significant difference between lasso and ridge regression [41]. Their simulations did not include a comparison to correlation networks, nor were there different topologies investigated, which clearly has a large impact on the results. In recent years, generalizations and variants of the lasso have been developed, among which the graphical lasso (the one in [41] is an approximation to the graphical lasso used here), which, together with the shrinkage estimator, turned out particularly suitable for large-scale network recovery in the present simulation scenario.

Our application to resting-state fMRI illustrated that partial correlation networks are more consistent and reliable than networks obtained from pairwise correlations. The inappropriateness of pairwise correlations to infer connectivity networks also holds for other areas of research, such as genetics [24]. Thus, we recommend the use of partial correlations obtained with the graphical lasso or shrinkage estimator to build large-scale networks.
